# Addressing climate change with behavioral science: A global intervention tournament in 63 countries

**DOI:** 10.1126/sciadv.adj5778

**Published:** 2024-02-07

**Authors:** Madalina Vlasceanu, Kimberly C. Doell, Joseph B. Bak-Coleman, Boryana Todorova, Michael M. Berkebile-Weinberg, Samantha J. Grayson, Yash Patel, Danielle Goldwert, Yifei Pei, Alek Chakroff, Ekaterina Pronizius, Karlijn L. van den Broek, Denisa Vlasceanu, Sara Constantino, Michael J. Morais, Philipp Schumann, Steve Rathje, Ke Fang, Salvatore Maria Aglioti, Mark Alfano, Andy J. Alvarado-Yepez, Angélica Andersen, Frederik Anseel, Matthew A. J. Apps, Chillar Asadli, Fonda Jane Awuor, Flavio Azevedo, Piero Basaglia, Jocelyn J. Bélanger, Sebastian Berger, Paul Bertin, Michał Białek, Olga Bialobrzeska, Michelle Blaya-Burgo, Daniëlle N. M. Bleize, Simen Bø, Lea Boecker, Paulo S. Boggio, Sylvie Borau, Björn Bos, Ayoub Bouguettaya, Markus Brauer, Cameron Brick, Tymofii Brik, Roman Briker, Tobias Brosch, Ondrej Buchel, Daniel Buonauro, Radhika Butalia, Héctor Carvacho, Sarah A. E. Chamberlain, Hang-Yee Chan, Dawn Chow, Dongil Chung, Luca Cian, Noa Cohen-Eick, Luis Sebastian Contreras-Huerta, Davide Contu, Vladimir Cristea, Jo Cutler, Silvana D'Ottone, Jonas De Keersmaecker, Sarah Delcourt, Sylvain Delouvée, Kathi Diel, Benjamin D. Douglas, Moritz A. Drupp, Shreya Dubey, Jānis Ekmanis, Christian T. Elbaek, Mahmoud Elsherif, Iris M. Engelhard, Yannik A. Escher, Tom W. Etienne, Laura Farage, Ana Rita Farias, Stefan Feuerriegel, Andrej Findor, Lucia Freira, Malte Friese, Neil Philip Gains, Albina Gallyamova, Sandra J. Geiger, Oliver Genschow, Biljana Gjoneska, Theofilos Gkinopoulos, Beth Goldberg, Amit Goldenberg, Sarah Gradidge, Simone Grassini, Kurt Gray, Sonja Grelle, Siobhán M. Griffin, Lusine Grigoryan, Ani Grigoryan, Dmitry Grigoryev, June Gruber, Johnrev Guilaran, Britt Hadar, Ulf J.J. Hahnel, Eran Halperin, Annelie J. Harvey, Christian A. P. Haugestad, Aleksandra M. Herman, Hal E. Hershfield, Toshiyuki Himichi, Donald W. Hine, Wilhelm Hofmann, Lauren Howe, Enma T. Huaman-Chulluncuy, Guanxiong Huang, Tatsunori Ishii, Ayahito Ito, Fanli Jia, John T. Jost, Veljko Jovanović, Dominika Jurgiel, Ondřej Kácha, Reeta Kankaanpää, Jaroslaw Kantorowicz, Elena Kantorowicz-Reznichenko, Keren Kaplan Mintz, Ilker Kaya, Ozgur Kaya, Narine Khachatryan, Anna Klas, Colin Klein, Christian A. Klöckner, Lina Koppel, Alexandra I. Kosachenko, Emily J. Kothe, Ruth Krebs, Amy R. Krosch, Andre P.M. Krouwel, Yara Kyrychenko, Maria Lagomarsino, Claus Lamm, Florian Lange, Julia Lee Cunningham, Jeffrey Lees, Tak Yan Leung, Neil Levy, Patricia L. Lockwood, Chiara Longoni, Alberto López Ortega, David D. Loschelder, Jackson G. Lu, Yu Luo, Joseph Luomba, Annika E. Lutz, Johann M. Majer, Ezra Markowitz, Abigail A. Marsh, Karen Louise Mascarenhas, Bwambale Mbilingi, Winfred Mbungu, Cillian McHugh, Marijn H.C. Meijers, Hugo Mercier, Fenant Laurent Mhagama, Katerina Michalakis, Nace Mikus, Sarah Milliron, Panagiotis Mitkidis, Fredy S. Monge-Rodríguez, Youri L. Mora, David Moreau, Kosuke Motoki, Manuel Moyano, Mathilde Mus, Joaquin Navajas, Tam Luong Nguyen, Dung Minh Nguyen, Trieu Nguyen, Laura Niemi, Sari R. R. Nijssen, Gustav Nilsonne, Jonas P. Nitschke, Laila Nockur, Ritah Okura, Sezin Öner, Asil Ali Özdoğru, Helena Palumbo, Costas Panagopoulos, Maria Serena Panasiti, Philip Pärnamets, Mariola Paruzel-Czachura, Yuri G. Pavlov, César Payán-Gómez, Adam R. Pearson, Leonor Pereira da Costa, Hannes M. Petrowsky, Stefan Pfattheicher, Nhat Tan Pham, Vladimir Ponizovskiy, Clara Pretus, Gabriel G. Rêgo, Ritsaart Reimann, Shawn A. Rhoads, Julian Riano-Moreno, Isabell Richter, Jan Philipp Röer, Jahred Rosa-Sullivan, Robert M. Ross, Anandita Sabherwal, Toshiki Saito, Oriane Sarrasin, Nicolas Say, Katharina Schmid, Michael T. Schmitt, Philipp Schoenegger, Christin Scholz, Mariah G. Schug, Stefan Schulreich, Ganga Shreedhar, Eric Shuman, Smadar Sivan, Hallgeir Sjåstad, Meikel Soliman, Katia Soud, Tobia Spampatti, Gregg Sparkman, Ognen Spasovski, Samantha K. Stanley, Jessica A. Stern, Noel Strahm, Yasushi Suko, Sunhae Sul, Stylianos Syropoulos, Neil C. Taylor, Elisa Tedaldi, Gustav Tinghög, Luu Duc Toan Huynh, Giovanni Antonio Travaglino, Manos Tsakiris, İlayda Tüter, Michael Tyrala, Özden Melis Uluğ, Arkadiusz Urbanek, Danila Valko, Sander van der Linden, Kevin van Schie, Aart van Stekelenburg, Edmunds Vanags, Daniel Västfjäll, Stepan Vesely, Jáchym Vintr, Marek Vranka, Patrick Otuo Wanguche, Robb Willer, Adrian Dominik Wojcik, Rachel Xu, Anjali Yadav, Magdalena Zawisza, Xian Zhao, Jiaying Zhao, Dawid Żuk, Jay J. Van Bavel

**Affiliations:** ^1^ Department of Psychology, New York University, New York, NY 10003, USA.; ^2^ Department of Cognition, Emotion, and Methods in Psychology, Faculty of Psychology, University of Vienna, Vienna 1010, Austria.; ^3^ Craig Newmark Center for Journalism Ethics and Security, Columbia University, New York, NY 10018, USA.; ^4^ Institute for Rebooting Social Media, Harvard University, Cambridge, MA 02138, USA.; ^5^ Department of Psychology, Stanford University, Stanford, CA 94305, USA.; ^6^ San Luis Obispo, CA 93405, USA.; ^7^ Copernicus Institute of Sustainable Development, Utrecht University, Utrecht, 3584 CB, Netherlands.; ^8^ Department of Psychology, University of Pittsburgh, Pittsburgh, PA 15260, USA.; ^9^ School of Public Policy and Urban Affairs, Northeastern University, Boston, MA 02115, USA.; ^10^ Department of Psychology, Northeastern University, Boston, MA 02115, USA.; ^11^ Amazon, Seattle, WA 98109, USA.; ^12^ Department of Psychology, Carl von Ossietzky University of Oldenburg, Oldenburg 26129, Germany.; ^13^ Santa Lucia Foundation, IRCCS, Rome 179, Italy.; ^14^ Department of Psychology, Sapienza University of Rome, Rome 185, Italy.; ^15^ Department of Philosophy, Macquarie University, Sydney, NSW 2000, Australia.; ^16^ Universidad Peruana Cayetano Heredia, San Martín de Porres 15102, Peru.; ^17^ Post-Graduation Program in Linguistics, Federal University of Paraná, Curitiba 80060150, Brasil.; ^18^ UNSW Business School, University of New South Wales, Sydney, NSW 2052, Australia.; ^19^ Centre for Human Brain Health, School of Psychology, University of Birmingham, Birmingham B15 2TT, UK.; ^20^ Psychology Scientific Research Institute, Baku, Azerbaijan.; ^21^ Kenya Marine and Fisheries Research Institute, Kisumu 1881-40100, Kenya.; ^22^ Department of Psychology, University of Groningen, Groningen 9712TS, Netherlands.; ^23^ Department of Economics, University of Hamburg, Hamburg 20146, Germany.; ^24^ Department of Psychology, New York University Abu Dhabi, Abu Dhabi 129188, United Arab Emirates.; ^25^ Department of Sociology, University of Bern, Bern 3012, Switzerland.; ^26^ LAPCOS, Université Côte d’Azur, Nice 6357, France.; ^27^ Center for Social and Cultural Psychology, Université libre de Bruxelles, Brussels 1050, Belgium.; ^28^ Institute of Psychology, Faculty of Historical and Pedagogical Sciences, University of Wroclaw, Wroclaw 50-120, Poland.; ^29^ Institute of Psychology, SWPS University, Warsaw 03-815, Poland.; ^30^ Department of Psychology, Division of Behavioral & Organizational Sciences, Claremont Graduate University, Claremont, NH 91711, USA.; ^31^ Behavioural Science Institute, Radboud University, Nijmegen, 6500 HE, Netherlands.; ^32^ Department of Strategy and Management, Norwegian School of Economics, Bergen 5045, Norway.; ^33^ Department of Economic Psychology, Social Psychology and Experimental Methods, Leuphana University Lüneburg, Lüneburg 21335, Germany.; ^34^ Social and Cognitive Neuroscience Laboratory, Mackenzie Presbyterian University, Sao Paulo 1241001, Brazil.; ^35^ Toulouse Business School, Institute for Advanced Study in Toulouse. Toulouse, 31000, France.; ^36^ Department of Economics, University of Hamburg, Hamburg 20146, Hamburg.; ^37^ School of Psychology, University of Birmingham, Birmingham B15 2TT, UK.; ^38^ Department of Psychology, University of Wisconsin–Madison, Madison, WI 53706, USA.; ^39^ Department of Psychology, University of Amsterdam, Amsterdam, 1018 WT, Netherlands.; ^40^ Department of Psychology, Inland Norway University of Applied Sciences, Elverum 2418, Norway.; ^41^ Policy Research Department, Kyiv School of Economics, Kyiv 2000, Ukraine.; ^42^ Department of Organisation, Strategy, and Entrepreneurship, School of Business and Economics, Maastricht University, Maastricht 6211 LK, Netherlands.; ^43^ Department of Psychology and Swiss Center for Affective Sciences, University of Geneva, Geneva 1205, Switzerland.; ^44^ Institute for Sociology of the Slovak Academy of Sciences, Slovak Academy of Sciences, Bratislava 81364, Slovakia.; ^45^ Psychological Science, Pomona College, Claremont, CA 91711, USA.; ^46^ Department of Movement Sciences, KU Leuven, Leuven 3001, Belgium.; ^47^ Escuela de Psicología, Pontificia Universidad Católica de Chile, Santiago, Chile.; ^48^ School of Psychology, Speech, and Hearing, University of Canterbury, University of Canterbury, Christchurch 8051, New Zealand.; ^49^ Department of Marketing, King’s Business School, King’s College London, London WC2B 4BG, UK.; ^50^ Department of Management and Marketing, University of Melbourne, Melbourne, VIC 3010, Australia.; ^51^ Department of Biomedical Engineering, Ulsan National Institute of Science and Technology, Ulsan 44919, Republic of Korea.; ^52^ Department of Marketing, University of Virginia, Darden School of Business, Charlottesville, VA 22903, USA.; ^53^ Department of Psychology, The Hebrew University, Jerusalem 9190501, Israel.; ^54^ Department of Psychology, University of Groningen, Groningen 9712 CP, Netherlands.; ^55^ Center for Social and Cognitive Neuroscience (CSCN), School of Psychology, Universidad Adolfo Ibáñez, Viña del Mar, Chile.; ^56^ Faculty of Management, Canadian University Dubai, Dubai 117781, United Arab Emirates.; ^57^ Kieskompas–Election Compass, Amsterdam 1052XH, Netherlands.; ^58^ Escuela de Psicología, Pontificia Universidad Católica de Chile, Santiago 8331150, Chile.; ^59^ Department of Developmental, Personality and Social Psychology, Ghent University, Ghent 9000, Belgium.; ^60^ Department of People Management and Organization, Esade Business School, Universitat Ramon Llull, Barcelona 8034, Spain.; ^61^ Behavioral Economics and Engineering Group, KU Leuven, Leuven 3000, Belgium.; ^62^ LP3C, Université Rennes 2, Rennes 35000, France.; ^63^ Department of Psychology, Saarland University, Saarbrücken 66123, Germany.; ^64^ Center for Earth System Research and Sustainability (CEN), University of Hamburg, Hamburg 20146, Germany.; ^65^ Amsterdam School of Communication Research, University of Amsterdam, Amsterdam 1018WV, Netherlands.; ^66^ Department of Psychology, University of Latvia, Riga, Latvia.; ^67^ Department of Management, Aarhus University, Aarhus 8210, Denmark.; ^68^ Department of Psychology, University of Birmingham, Birmingham B15 2TT, UK.; ^69^ Department of Vision Science, University of Leicester, Leicester LE1 7RH, UK.; ^70^ Department of Clinical Psychology, Utrecht University, Utrecht 3508 TC, Netherlands.; ^71^ Institute of Management & Organization, Leuphana University Lüneburg, Lüneburg 21335, Germany.; ^72^ Department of Political Science & Annenberg School for Communication, University of Pennsylvania, Philadelphia, PA 19104, USA.; ^73^ Department of Psychology, University of Salzburg, Salzburg 5020, Salzburg.; ^74^ HEI-Lab: Digital Human-Environment Interaction Labs, Lusófona University, Lisbon 1700, Portugal.; ^75^ School of Management, Ludwig Maximilian University of Munich, Munich 80539, Germany.; ^76^ Institute of European Studies and International Relations, Faculty of Social and Economic Sciences, Comenius University Bratislava, Bratislava 82105, Slovakia.; ^77^ Laboratorio de Neurociencia, Escuela de Negocios, Universidad Torcuato Di Tella, Buenos Aires C1428, Argentina.; ^78^ School of Global Studies, Thammasat University, Bangkok 12121, Thailand.; ^79^ Center for Sociocultural Research, HSE University, Moscow 101000, Russia.; ^80^ Environmental Psychology, Department of Cognition, Emotion, and Methods in Psychology, Faculty of Psychology, University of Vienna, Vienna A-1010, Austria.; ^81^ Institute for Management and Organization, Leuphana University Lüneburg, Lüneburg 21335, Germany.; ^82^ Macedonian Academy of Sciences and Arts, Skopje 1000, North Macedonia.; ^83^ Faculty of Philosophy, Institute of Psychology, Faculty of Philosophy, Jagiellonian University, Krakow 30-060, Poland.; ^84^ Jigsaw, Google, New York, NY 10011, USA.; ^85^ Harvard Business School, Harvard University, Boston, MA 2163, USA.; ^86^ Department of Psychology, Harvard University, Cambridge, MA 2138, USA.; ^87^ Digital Data and Design Institute at Harvard, Harvard University, Allston, Boston, MA 2134, USA.; ^88^ School of Psychology and Sport Science, Anglia Ruskin University, Cambridge CB1 1PT, UK.; ^89^ Psychosocial Science, University of Bergen, Bergen 5007, Norway.; ^90^ Cognitive and Behavioral Neuroscience Laboratory, University of Stavanger, Stavanger 4021, Norway.; ^91^ Department of Psychology and Neuroscience, University of North Carolina, Chapel Hill, Chapel Hill, NC 27599, USA.; ^92^ Department of Psychology, Ruhr University Bochum, Bochum 44801, Germany.; ^93^ Department of Psychology, University of Limerick, Limerick V94T9PX, Ireland.; ^94^ Department of Psychology, University of York, York YO10 5DD, UK.; ^95^ Department of Personality Psychology, Yerevan State University, Yerevan 0025, Armenia.; ^96^ Department of Psychology and Neuroscience, University of Colorado Boulder, Boulder, CO 80309, USA.; ^97^ Division of Social Sciences, University of the Philippines Visayas, Miagao 5023, Philippines.; ^98^ Baruch Ivcher School of Psychology, Reichman University, Herzliya 4610101, Israel.; ^99^ Faculty of Psychology, University of Basel, Basel 4055, Switzerland.; ^100^ Department of Psychology, University of Oslo, Oslo 373, Norway.; ^101^ Nencki Institute of Experimental Biology, Polish Academy of Sciences, Warsaw 02-093, Poland.; ^102^ School of Psychology, University of Sussex, Falmer BN1 9RH, UK.; ^103^ Anderson School of Management, University of California, Los Angeles, Los Angeles, CA 90095, USA.; ^104^ School of Economics & Management, Kochi University of Technology, Kami City 782-8502, Japan.; ^105^ School of Psychology, Speech and Hearing, University of Canterbury, Christchurch 8051, New Zealand.; ^106^ Department of Business Administration, University of Zurich, Zurich 8032, Switzerland.; ^107^ Department of Psychology, Universidad Nacional de San Antonio Abad del Cusco, Cusco 800, Peru.; ^108^ Department of Media and Communication, City University of Hong Kong, Kowloon 999077, Hong Kong, China.; ^109^ Department of Psychology, Japan Women’s University, Tokyo 1128681, Japan.; ^110^ Graduate School of Education, Tohoku University, Sendai 9808576, Japan.; ^111^ Department of Psychology, Seton Hall University, South Orange, NJ 7079, USA.; ^112^ Department of Psychology, Faculty of Philosophy, University of Novi Sad, Novi Sad 21000, Serbia.; ^113^ Doctoral School of Social Sciences, Nicolaus Copernicus University, Toruń 87-100, Poland.; ^114^ Green Dock, Hostivice 25301, Czech Republic.; ^115^ Faculty of Social Sciences, Tampere University, Tampere 33100, Finland.; ^116^ INVEST Research Flagship, University of Turku, Turku 20014, Finland.; ^117^ Institute of Security and Global Affairs, Leiden University, The Hague 2511DP, Netherlands.; ^118^ Erasmus School of Law, Erasmus University Rotterdam, Rotterdam 3062PA, Netherlands.; ^119^ Shamir Research Institute, University of Haifa, Haifa 3498838, Israel.; ^120^ Department of Learning and Instructional Sciences, University of Haifa, Haifa, 3498838, Israel.; ^121^ Deparment of Economics, American University of Sharjah, Sharjah 26666, United Arab Emirates.; ^122^ School of Psychology, Deakin University, Geelong, VIC 3216, Australia.; ^123^ School of Philosophy, Australian National University, Canberra, ACT 2600, Australia.; ^124^ Department of Psychology, Norwegian University of Science and Technology, Trondheim 7049, Norway.; ^125^ Department of Management and Engineering, Linköping University, Linköping 58183, Sweden.; ^126^ Academic and Research Laboratory of Neurotechnology, Ural Federal University, Ekaterinburg 620075, Russia.; ^127^ Department of Experimental Psychology, Ghent University, Ghent 9000, Belgium.; ^128^ Department of Psychology, Cornell University, Ithaca, NY 14850, USA.; ^129^ Departments of Political Science and Communication Science, Vrije Universiteit Amsterdam, Amsterdam 1081 HV, Netherlands.; ^130^ Department of Psychology, University of Cambridge, Cambridge CB2 3EL, UK.; ^131^ Psychology of Sustainability and Behavior Change, University of Basel, Basel 4055, Switzerland.; ^132^ Management & Organizations, Stephen M. Ross School of Business, University of Michigan, Ann Arbor, MI 48105, USA.; ^133^ John E. Walker Department of Economics, Clemson University, Clemson, SC 29634, USA.; ^134^ Andlinger Center for Energy and the Environment, Princeton University, Princeton, NJ 8544, USA.; ^135^ School of Business and Creative Industries, University of the Sunshine Coast, Queensland, BNE 4556, Australia.; ^136^ Department of Philosophy, Macquarie University, Sydney, NSW 2109, Australia.; ^137^ Department of Marketing, Bocconi University, Milan 20136, Italy.; ^138^ Department of Communication Science, Vrije Universiteit Amsterdam, Amsterdam 1081 HV, Netherlands.; ^139^ Institute of Management and Organization, Leuphana University of Lüneburg, Lueneburg 21337, Germany.; ^140^ MIT Sloan School of Management, Massachusetts Institute of Technology, Cambridge, MA 2139, USA.; ^141^ Department of Psychology, University of British Columbia, Vancouver, BC V6T 1Z4, Canada.; ^142^ Tanzanian Fisheries Research Institute, Mwanza, Tanzania.; ^143^ Department of Psychology, Simon Fraser University, Burnaby, BC V5A 1S6, Canada.; ^144^ Department of Social, Organizational, & Economic Psychology, University of Hildesheim, Hildesheim 31141, Germany.; ^145^ Department of Environmental Conservation, University of Massachusetts Amherst, Amherst, MA 1003, USA.; ^146^ Department of Psychology, Georgetown University, Washington, DC 20057, USA.; ^147^ Research Centre for Greenhouse Gas Innovation (RCGI), University of São Paulo, São Paulo 05508-030, Brazil.; ^148^ Department of Social Psychology, Institute of Psychology, University of São Paulo, São Paulo 05508-030, Brazil.; ^149^ National Fisheries Resources Research Institute, Jinja, Uganda.; ^150^ Department of Civil and Water Resources Engineering School of Engineering and Technology, Sokoine University of Agriculture, Morogoro, Tanzania.; ^151^ Department of Psychology, University of Limerick, Limerick V94 T9PX, Ireland.; ^152^ Department of Communication Science, University of Amsterdam, Amsterdam 1001 NG, Netherlands.; ^153^ Institut Jean Nicod, Département d’études cognitives, ENS, EHESS, PSL University, CNRS, Paris 75005, France.; ^154^ Tanzania Fisheries Research Institute, Mwanza, Tanzania.; ^155^ Royal Holloway, University of London, Egham TW200EX, UK.; ^156^ School of Culture and Society–Interacting Minds Centre, Aarhus University, Aarhus 8000, Denmark.; ^157^ Neuropsychopharmacology and Biopsychology Unit, University of Vienna, Vienna 1010, Austria.; ^158^ Department of Psychology, Universidad Peruana Cayetano Heredia, Lima 2002, Peru.; ^159^ Fonds de la Recherche Scientifique, Brussels 1050, Belgium.; ^160^ Center for Social and Cultural Psychology, Université libre de Bruxelles, Brussels 1312, Belgium.; ^161^ School of Psychology, University of Auckland, Auckland 1010, New Zealand.; ^162^ Department of Management, The University of Tokyo, Tokyo 113-8654, Japan.; ^163^ Department of Psychology, University of Cordoba, Cordoba 14071, Spain.; ^164^ Département d’études cognitives, Institut Jean Nicod ENS, EHESS, PSL University, CNRS, Tokyo 113-8654, Japan.; ^165^ Comisión Nacional de Investigaciones Científicas y Técnicas (CONICET), Buenos Aires, Argentina.; ^166^ Laboratorio de Neurociencia, Escuela de Negocios, Universidad Torcuato Di Tella, Buenos Aires C1428 CABA, Argentina.; ^167^ University of Economics HCMC (UEH), Ho Chi Minh City, Vietnam.; ^168^ College of Management, National Kaohsiung University of Science and Technology, Kaohsiung 800, Taiwan.; ^169^ Department of Psychology and Dyson School of Applied Economics and Management, Cornell University, Ithaca, NY 14850, USA.; ^170^ Environmental Psychology, Department of Cognition, Emotion, and Methods in Psychology, Faculty of Psychology, University of Vienna, Vienna 1010, Austria.; ^171^ Department of Clinical Neuroscience, Karolinska Institutet, Stockholm 17177, Sweden.; ^172^ Department of Psychology, Stockholm University, Stockholm 11419, Sweden.; ^173^ Department of Psychology and Behavioural Sciences, Aarhus University, Aarhus 8000, Denmark.; ^174^ Department of Psychology, Kadir Has University, İstanbul 34083, Turkey.; ^175^ Department of Psychology, Marmara University, İstanbul 34722, Turkey.; ^176^ Department of Psychology, Üsküdar University, İstanbul 34662, Turkey.; ^177^ Department of Economics and Business, Universitat Pompeu Fabra, Barcelona 8005, Spain.; ^178^ Department of Political Science, Northeastern University, Boston, MA 2115, USA.; ^179^ IRCCS, Santa Lucia Foundation, Rome 142, Italy.; ^180^ Department of Clinical Neuroscience, Division of Psychology, Karolinska Institutet, Stockholm 171 77, Sweden.; ^181^ Penn Center for Neuroaesthetics, University of Pennsylvania, Philadelphia, PA 19104, USA.; ^182^ Institute of Psychology, University of Silesia in Katowice, Katowice 40-007, Poland.; ^183^ Institute of Medical Psychology and Behavioral Neurobiology, University of Tuebingen, Tuebingen 72076, Germany.; ^184^ Dirección Académica, Universidad Nacional de Colombia, Sede de La Paz, Cesar, Colombia.; ^185^ HEI-Lab: Digital Human-Environment Interaction Labs, Lusófona University, Lisbon, Portugal.; ^186^ Institute of Management and Organization, Leuphana University Lueneburg, Lueneburg 21337, Germany.; ^187^ School of Business, International University, Vietnam National University HCMC, Ho Chi Minh City 700000, Vietnam.; ^188^ Department of Psychobioloogy and Methodology of Heath Sciences, Universitat Autònima de Barcelona, Barcelona 8193, Spain.; ^189^ Center for Health and Biological Sciences, Mackenzie Presbyterian University, São Paulo 01221-040, Brazil.; ^190^ Department of Psychology, Georgetown University, Washington DC, 20057, USA.; ^191^ Center for Computational Psychiatry, Icahn School of Medicine at Mount Sinai, New York, NY 10029, USA.; ^192^ Faculty of Medicine, Universidad Cooperativa de Colombia, Villavicencio, Colombia.; ^193^ Department of Psychology, Faculty for Social and Educational Sciences, Norwegian University of Science and Technology, Trondheim 7491, Norway.; ^194^ Department of Psychology and Psychotherapy, Witten/Herdecke University, Witten 58455, Germany.; ^195^ Department of Psychology, University of California, Los Angeles, Los Angeles, CA 90095, USA.; ^196^ Department of Psychological and Behavioural Science, London School of Economics and Political Science, London WC2A 2AE, UK.; ^197^ Japan Society for the Promotion of Science, Tokyo 1020083, Japan.; ^198^ Faculty of Science and Engineering, Waseda University, Tokyo 1658555, Japan.; ^199^ Institute of Psychology, University of Lausanne, Lausanne 1015, Switzerland.; ^200^ Department of Management, Prague University of Economics and Business, Prague 13067, Czech Republic.; ^201^ Department of People Management and Organization, Universitat Ramon Llull, Esade Business School, Barcelona 8034, Spain.; ^202^ School of Economics & Finance, University of St Andrews, St Andrews KY16 9AJ, UK.; ^203^ School of Philosophical, Anthropological and Film Studies, University of St Andrews, St Andrews KY16 9AJ, UK.; ^204^ Department of Communication, Amsterdam School of Communication Research, University of Amsterdam, Amsterdam 1018WV, Netherlands.; ^205^ Department of Psychology, Widener University, Chester 19013, USA.; ^206^ Department of Cognitive Psychology, Universität Hamburg, Hamburg 20146, Germany.; ^207^ Department of Nutritional Sciences, University of Vienna, Vienna 1090, Austria.; ^208^ Harvard Business School, Harvard University, Boston, MA 2163, USA.; ^209^ Department of Social Psychology, Reichman University (RUNI), Herzliya 4610101, Israel.; ^210^ Research Center for Digital Transformation, Leuphana University Lüneburg, Lüneburg 21335, Germany.; ^211^ Department of Biomedicine, Aarhus University, Aarhus 8000, Denmark.; ^212^ Danish Research Institute of Translational Neuroscience (DANDRITE), Aarhus University, Aarhus 8000, Denmark.; ^213^ Faculty of Psychology and Educational Sciences, University of Geneva, Geneva 1205, Switzerland.; ^214^ Swiss Center for Affective Sciences, University of Geneva, Geneva 1205, Switzerland.; ^215^ Department of Psychology and Neuroscience, Boston College, Chestnut Hill, MA 2467, USA.; ^216^ Faculty of Philosophy, Ss. Cyril and Methodius University in Skopje, Skopje 1000, Republic of North Macedonia.; ^217^ Faculty of Philosophy, University of Ss. Cyril and Methodius in Trnava, Trnava 917 01, Slovakia.; ^218^ School of Medicine and Psychology, Australian National University, Canberra, ACT 200, Australia.; ^219^ Department of Psychology, University of Virginia, Charlottesville, VA 22902, USA.; ^220^ Faculty of Social Sciences/Psychology, Tampere University, Tampere FI-33014, Finland.; ^221^ Department of Psychology, Pusan National University, Busan 46241, Republic of Korea.; ^222^ Psychology and Neuroscience, Schiller Institute for Integrated Science and Society, Boston College, Brighton, MA 2135, USA.; ^223^ UQ Business School, University of Queensland, Brisbane, QLD 4067, Australia.; ^224^ Department of Developmental Psychology and Socialisation, University of Padova, Padua 35131, Italy.; ^225^ School of Business and Management, Queen Mary University of London, London E1 4NS, UK.; ^226^ Institute for the Study of Power, Crime, and Society | Department of Law & Criminology, Royal Holloway, University of London, Egham TW200EX, UK.; ^227^ Department of Psychology, Üsküdar University, Istanbul 34664, Turkey.; ^228^ Department of Public and International Affairs, City University of Hong Kong, Kowloon 999077, Hong Kong.; ^229^ School of Psychology, University of Sussex, Falmer BN19QH, UK.; ^230^ Institute of Pedagogy, Faculty of Historical and Pedagogical Sciences, University of Wroclaw, Wroclaw 50-120, Poland.; ^231^ Research Department, The South Ural University of Technology, Chelyabinsk 454052, Russia.; ^232^ Laboratory of Interdisciplinary Space Studies, School for Environmental and Social Studies, Tyumen State University, Tyumen 625003, Russia.; ^233^ Department of Psycholgy, Cambridge University, Cambridge CB2 3 EB, UK.; ^234^ Department of Medical and Clinical Psychology, Tilburg University, Tilburg 5037 AB, Netherlands.; ^235^ Behavioural Science Institute, Radboud University, Nijmegen 6500 HE, Netherlands.; ^236^ Department of Psychology, University of Latvia, Riga 1083, Latvia.; ^237^ Division of Psychology, Linköping University, Linköping 58183, Sweden.; ^238^ Department of Marketing Communication and Public Relations, Charles University, Prague 11000, Czech Republic.; ^239^ Kenya Marine and Fisheries Research Institute, Kisumu 1881-40100, Kenya.; ^240^ Department of Sociology, Stanford University, Stanford, CA 94305, USA.; ^241^ Faculty of Philosophy and Social Sciences, Nicolaus Copernicus University, Toruń 87-100, Poland.; ^242^ Department of Humanities and Social Sciences, Climate and Energy Policy Research Lab, Indian Institute of Technology Kanpur, Kanpur 208016, India.; ^243^ School of Computing, Engineering and Mathematical Sciences, La Trobe University Melbourne, Melbourne, VIC 3086, Australia.; ^244^ Kellogg School of Management, Northwestern University, Evanston, IL 60208, USA.; ^245^ Institute for Resources, Environment and Sustainability, University of British Columbia, Vancouver, BC V6T 1Z4, Canada.; ^246^ Faculty of Psychology, University of Warsaw, Warsaw 00-183, Poland.; ^247^ Center for Neural Science, New York University, New York, NY 10003, USA.

## Abstract

Effectively reducing climate change requires marked, global behavior change. However, it is unclear which strategies are most likely to motivate people to change their climate beliefs and behaviors. Here, we tested 11 expert-crowdsourced interventions on four climate mitigation outcomes: beliefs, policy support, information sharing intention, and an effortful tree-planting behavioral task. Across 59,440 participants from 63 countries, the interventions’ effectiveness was small, largely limited to nonclimate skeptics, and differed across outcomes: Beliefs were strengthened mostly by decreasing psychological distance (by 2.3%), policy support by writing a letter to a future-generation member (2.6%), information sharing by negative emotion induction (12.1%), and no intervention increased the more effortful behavior—several interventions even reduced tree planting. Last, the effects of each intervention differed depending on people’s initial climate beliefs. These findings suggest that the impact of behavioral climate interventions varies across audiences and target behaviors.

## Teaser

Climate interventions increase beliefs, policy support, and willingness to share information but not higher effort action.

## INTRODUCTION

The climate crisis is one of humanity’s most consequential and challenging problems (*1*). Successfully rising to the challenge depends on both “top-down” structural changes (e.g., regulation and investment) and “bottom-up” changes (e.g., individuals’ and collectives’ beliefs and behaviors). These bottom-up processes require widespread belief in climate change, support for climate change policy, and willingness to engage in climate action (*2*–*4*). The behavioral sciences have been seen as a crucial component in promoting bottom-up change, through the development of large-scale interventions that can shift public opinion and enable and support top-down governmental climate policies (*5*–*7*). However, it is unclear which strategies are most likely to motivate people to change their climate change beliefs and climate mitigation behaviors. Here, we assess the effectiveness of expert-crowdsourced, theoretically derived interventions at promoting a range of climate change mitigation behaviors in a large and diverse global sample.

A growing body of research across the behavioral sciences has been investigating intervention strategies aimed at boosting sustainable intentions and behaviors such as recycling, public transportation use, and household energy saving (*3*, *8*, *9*). For instance, communications aimed at reducing the psychological distance of climate change, by making it feel more geographically, socially, and temporally close, were effective at increasing climate concern and amplifying self-reported intentions to engage in mitigating behaviors, such as reducing energy consumption (*10*). Similarly, normative appeals that include an invitation to work together and “join in” were found effective at influencing behaviors such as charitable giving (*11*). These are only two examples in a growing list of behavioral interventions designed to mitigate climate change. Hence, there are numerous competing theories in the behavioral sciences about how to stimulate climate change beliefs and proenvironmental behaviors.

While many of these theories, as well as their corresponding interventions, are promising, they have been tested independently with different samples and, on separate outcomes, making it impossible to directly compare their effectiveness. In addition, assessing interventions on a single outcome renders it difficult to understand their effects on multiple facets of climate mitigation, which are all necessary to substantially reduce climate change (e.g., support for climate mitigation policy and sustainable behavior). These limitations are a major barrier to resolving theoretical debates within the scientific community (*12*, *13*) and to translating scientific findings into impactful policies (*14*, *15*). Moreover, traditional attempts to compare interventions (e.g., meta-analyses) (*16*) are limited by differences in experimental protocols, outcome variables, samples, and operationalizations (*17*, *18*, *19*). These differences hinder evaluations of the relative effectiveness of different theories and interventions (*15*). To address these concerns, we used the megastudy approach—an experimental paradigm similar to a randomized controlled trial but designed to evaluate the efficacy of many interventions on several outcome variables, in the same large-scale experiment (*18*). This provides a rigorous direct comparison of competing approaches to climate change mitigation.

Another challenge is that most prior work across the behavioral sciences (including the megastudy approach) has been mainly conducted on Western, educated samples from industrialized, rich, and developed countries (i.e., WEIRD) (*20*). Results from these samples may not generalize to other nations, restricting the ability to apply findings beyond WEIRD populations. This is a particular problem for a topic like climate change where the social and political dynamics, as well as exposure to the impacts of climate change, vary across countries (*21*, *22*). While wealthier nations are disproportionately responsible for causing climate change (*23*), it is still important to understand which interventions work across a diversity of cultures since the most effective mitigation strategies will likely require global cooperation. Accordingly, we leveraged the many labs approach, in which the same study is being conducted by many research laboratories around the world, aggregating the results in the same international dataset (*17*, *24*).

**Fig. 1.**
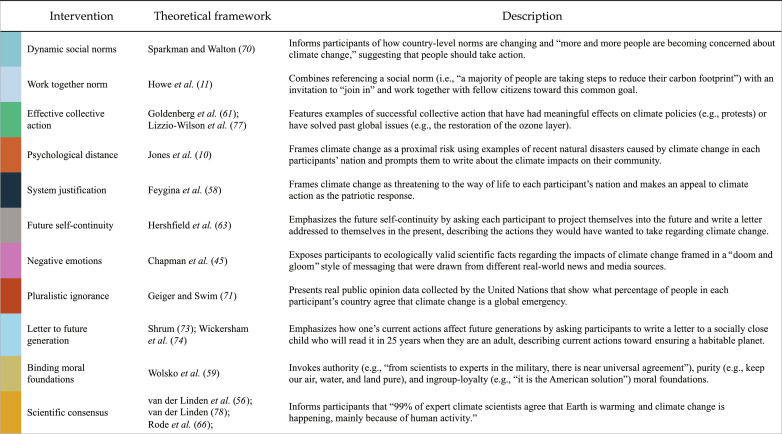
Interventions, theoretical frameworks, and brief descriptions.

In this global megastudy, we crowdsourced interventions previously found to stimulate climate mitigation from behavioral science experts (fig. S5). We used a crowdsourcing approach to determine which interventions to test, given recent evidence that crowdsourcing can improve the quality of scientific investigations by promoting ideation, inclusiveness, transparency, rigor, and reliability (*25*). This resulted in the identification of 11 behavioral interventions based on competing theoretical frameworks in the behavioral sciences (Fig. 1).

We tested these interventions in a global tournament spanning 63 countries on four outcome variables, which were also crowdsourced and selected on the basis of their theoretical and practical relevance to climate mitigation. The first outcome on which we assessed each intervention was belief in climate change (four items; e.g., “Climate change poses a serious threat to humanity”). Given that belief is a key antecedent of proenvironmental intentions, behavior, and policy support (*26*), we examined how the interventions would affect these outcomes for different people along the belief continuum ranging from skeptics to true believers.

The second outcome was support for climate change mitigation policy (nine items; e.g., “I support raising carbon taxes on gas/fossil fuels/coal”). Given that successful climate mitigation requires large-scale policy reform (*1*) and the public’s support for climate policies is the top predictor of policy adoption (*27*), this outcome variable reflects the importance of impactful systemic change, rather than private mitigation efforts based on individual decision-making (*28*–*30*). Recent work argues that individual-level behaviors should be targeted alongside structural changes (*31*), especially since framing climate change as an individual level problem can backfire, leading to feelings of helplessness and concerns about free riding (*32*, *33*).

To target more ecologically valid behavior and climate activism (*34*), the third outcome was willingness to share climate mitigation information on social media (i.e., “Did you know that removing meat and dairy for only two out of three meals per day could decrease food-related carbon emissions by 60%?”). While this behavior is relatively low effort, recent work suggests climate information sharing with one’s community as an essential step in addressing the climate crisis (*35*).

Last, given the large gap between self-reported measures and objective proenvironmental behavior (*36*), the fourth outcome we targeted was a more effortful behavior of contributing to a real tree planting initiative by engaging in a cognitively demanding task (i.e., a modified version of the work for environmental protection task or WEPT) (*37*). The WEPT is a multitrial, web-based procedure in which participants choose to exert voluntary effort screening stimuli for specific numerical combinations (i.e., an even first digit and odd second digit) in exchange for donations to a tree-planting environmental organization. Thus, they had the opportunity to produce actual environmental benefits at actual behavioral costs, mimicking classic sustainable behavior trade-offs (*38*–*40*).

Participants (*N* = 59,440, from 63 counties; Table 1) were mostly recruited through online data collection platforms (80.8%) or via convenience/snowball sampling (19.1%; Table 1). They were randomly assigned to 1 of 11 experimental interventions (Fig. 1) or a no intervention control condition in which they read a passage from a literary text. Then, in a randomized order, participants indicated their climate beliefs, climate policy support, and willingness to share climate-related information on social media. Last, participants were able to opt into completing up to eight pages of a tree-planting task, each completed page resulting in the real planting of a tree through a donation to The Eden Reforestation Project. As a result of participants’ behavior, our team actually planted 333,333 trees. Assuming that the average fully grown tree absorbs between 10 and 40 kg of carbon dioxide per year in 5 to 10 years when all trees are fully grown, the efforts from this project will result in ~9,999,990 kg of carbon dioxide sequestered per year, which is the equivalent amount of carbon dioxide used to produce energy for 1260 U.S. homes.

**Table 1.** Variables on which the samples in each country were matched to the population. Countries in which no demographic variable was census matched are marked as “N/A” in the “matched variables” column. SES, socioeconomic status.

Sample	Matched variables	*N*	Sample	Matched variables	*N*
Algeria	N/A	528	Philippines	N/A	145
Armenia	N/A	492	Poland_1	Age, gender, education	1883
Australia	Gender	979	Poland_2	N/A	463
Austria	Age, gender	502	Portugal	N/A	499
Belgium_1	Age, gender	522	Romania	N/A	411
Belgium_2	Age, gender	512	Russia_1	N/A	718
Brazil	Age, gender, education	1261	Russia_2	Region, ethnicity	395
Bulgaria	Age, gender	778	Russia_3	N/A	322
Canada_1	N/A	858	Saudi Arabia	N/A	489
Canada_2	Age, gender	303	Serbia	N/A	337
Chile	Age, gender, region, SES	1992	Singapore	N/A	500
China	N/A	896	Slovakia	Age, gender, region, municipality size	1027
Czechia	N/A	547	Slovenia	Age, gender	501
Denmark	Age, gender, region	792	South Africa	Age, gender	496
Ecuador	Age, gender, region	679	South Korea	Age, gender	639
Finland	Age, gender	625	Spain_1	N/A	110
France	Age, gender	1480	Spain_2	Age, gender, region	434
Gambia	N/A	527	Sri Lanka	N/A	413
Germany	Age, gender, region	1545	Sudan	Age, gender	623
Ghana	Age, gender	522	Sweden	Age, gender	2393
Greece	Age, gender	597	Switzerland_1	Age, gender	512
India	N/A	688	Switzerland_2	Age, gender	531
Ireland	N/A	753	Taiwan	N/A	206
Israel	Age, gender, region, ethnicity	1384	Tanzania	Age, gender	104
Italy_1	Age, gender, region	591	Thailand	N/A	586
Italy_2	Gender	993	Turkey_1	N/A	359
Japan_1	N/A	653	Turkey_2	Age, gender	347
Japan_2	Income, education, region, ethnicity	802	Uganda	Age, gender	476
Kenya	Age, gender	409	UK_1	N/A	220
Latvia	Income, education, ethnicity	485	UK_2	Age, gender	952
Mexico	Age, gender	490	UK_3	N/A	234
Morocco	Age, gender	474	UK_4	gender	501
Netherlands_1	Age, gender	854	Ukraine	N/A	496
Netherlands_2	Age, gender	510	UAE	Broadly representative	554
Netherlands_3	N/A	500	Uruguay	N/A	838
New Zealand	Gender	1005	USA_1	Age, gender	2360
Nigeria	Age, gender	1513	USA_2	Age, gender, region, ethnicity	5055
North Macedonia	N/A	878	USA_3	Age, gender	497
Norway	Age, gender, ethnicity	997	Venezuela	N/A	110
Peru	Age, gender	405	Vietnam	N/A	383

## RESULTS

### Main effects of intervention

**Fig. 2.**
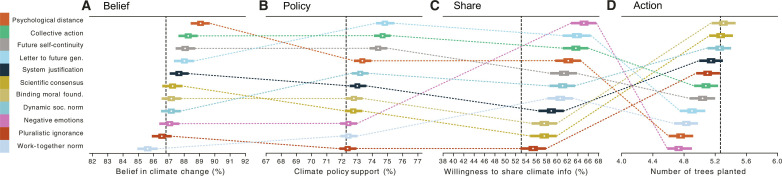
Average effects (i.e., posterior estimates using Bayesian regressions) by intervention for each outcome. Dots indicate the mean, with error bars indicating the 94% credible region. Thicker error bars indicate the interquartile range. Vertical lines indicate control average. (**A**) Belief, (**B**) support for policy, (**C**) willingness to share climate change information on social media, and (**D**) number of trees planted in the WEPT. Estimates are reported in tables S1 to S4.

First, we examined the effect of each intervention on each of the four outcomes, estimated using a series of Bayesian regressions (see Materials and Methods). As the goal of this study is to estimate the relative effectiveness of treatments, in contrast to establishing non-null effects or differences, Bayesian estimation is preferable to classical null hypothesis significance testing. Bayesian techniques produce posterior distributions for parameters (here, treatment effects) that characterize their magnitude and associated uncertainty. We summarize this distribution in Fig. 2 using a point estimate corresponding to the mean and a 94% credible region, which differs from a confidence interval in which it indicates a region with a 94% chance of containing the unobserved parameter value (*41*). Moreover, we also conducted similar frequentist analyses (hierarchical mixed models) and found converging results (see the Supplementary Materials for details).

We began by assessing the main intervention effects on each outcome. For belief in climate change (measured on a scale from 0 to 100), the top performing intervention, decreasing psychological distance, increased beliefs by an absolute effect size of 2.3% (1.6 to 2.9) (94% credible region) compared to the control condition. Consistent with prior work (*10*), some interventions slightly increased beliefs. However, other interventions had near-zero effect, suggesting that findings of some prior research did not extend to this context (Fig. 2A) (*11*).

For climate policy support (measured on a scale from 0 to 100), the intervention with the largest average effect was writing a letter to a member of the future generation, which increased policy support by 2.6% (2.0 to 3.2). Similar to belief, all interventions produced either more policy support or no discernible differences from the control condition (Fig. 2B).

For willingness to share climate change information on social media (measured as a binary choice), all interventions generally increased intentions to share. The largest gains were exhibited in the negative emotion induction condition, which led to 12.1% (9.8 to 14.6) more sharing compared to the control condition (Fig. 2C).

For the number of pages completed on the WEPT tree-planting task (from 0 to 8), no intervention was better than the control condition, and some interventions (i.e., decreasing psychological distance, inducing negative emotions, work-together normative appeals, and writing a letter to a future-generation member) appeared to reduce tree planting (Fig. 2D). These results held regardless of the operationalization of a tree planted as participants’ confirmation that they wanted to complete another WEPT page or their accuracy in the task (table S24).

The interventions that produced negative effects on the WEPT were also those that took the most time to complete (see the Supplementary Materials). Assuming that participants have a limited budget of time for completing surveys and given that the tree-planting task requires time, it is expected that we observed a trade-off between the time spent on the intervention and on the outcome task. Therefore, in an exploratory analysis (tables S22 and S23), we assessed the effects of the interventions when adjusting for the time spent on each intervention. While we still observed the negative effects of some interventions on tree planting, we now also observed positive effects of five interventions. That is, when controlling for intervention length, binding moral foundations, scientific consensus, dynamic norms, pluralistic ignorance, and system justification all increased the number of trees planted compared to the control condition. Thus, in the absence of time constraints, these interventions might increase proenvironmental behavior. However, the degree to which these findings actually generalize to proenvironmental behaviors that do not hinge on time (e.g., donations) should be assessed in future studies.

For further assessing the average effects of each intervention on each outcome within any subsample of interest varying along demographics such as nationality, political ideology, age, gender, education, or income level, we provide an easy to use and disseminate web tool: https://climate-interventions.shinyapps.io/climate-interventions/.

### Heterogeneous intervention effects along initial belief continuum

**Fig. 3.**
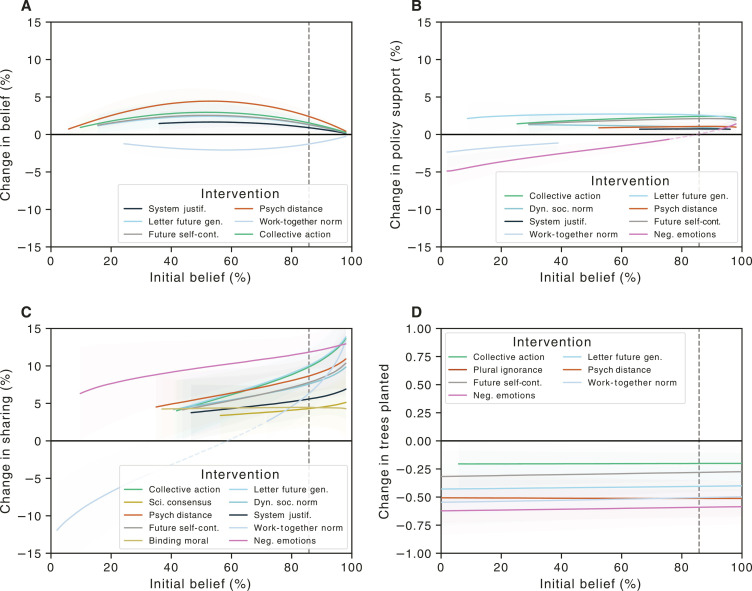
Marginal effects (as the difference between interventions and control) as a function of estimated preintervention belief in climate change. Lines indicate the average effect size, with shaded regions indicating the 94% credible region. For visual clarity, regions in which the 94% credible region overlap zero are omitted from the figure. Where interventions have positive and negative effects that meet these criteria, a dashed line is used to connect these regions. The dashed vertical line indicates average belief, where effects in Fig. 1 are estimated. (**A**) Climate change belief, (**B**) policy support, (**C**) sharing information on social media, and (**D**) trees planted via the WEPT.

We found a high level of belief in climate change [i.e., 85.7% (85.2 to 86.2), an estimate computed using the ratings of belief in the control participants and estimated preintervention levels of belief from all other participants]. This could raise two potential concerns when evaluating the main effects of the interventions mentioned above: On the one hand, at this high level of belief, participants may be particularly receptive to interventions. As a result, average effects may tend to overestimate the effectiveness of interventions in applied contexts where the aim is to increase belief or policy support in skeptical participants that do not already believe in climate change. On the other hand, as our outcomes are bounded, these high levels of belief may lead to ceiling effects in the estimation of the average effects, which may undervalue the true effectiveness of the interventions. To address this concern, we conducted an additional analysis where we modeled heterogeneous effects as a function of unobserved preintervention belief (see Materials and Methods and the Supplementary Materials). This analysis allowed us to visualize how effective interventions were across the continuum from climate change skeptics (i.e., those with initial beliefs less than 35%) to true believers (i.e., those with initial beliefs higher than 65%; Fig. 3).

For the impact of interventions on belief (Fig. 3A), we found clear indications of ceiling effects with many interventions being maximally impactful among uncertain participants, even those with low to moderate levels of initial belief. Even in participants with low levels of preexisting climate change belief (i.e., less than 35%), interventions such as reducing psychological distance, future self-continuity, and effective collective action are all viable ways to increase belief in climate change.

For policy support, a different pattern emerged. Interventions such as writing a letter to a member of the future generation, collective action efficacy, future self-continuity, and decreasing psychological distance all increased support for climate policy (Fig. 3B). Those same interventions appear to function well on individuals with modest to high levels of initial climate change belief (i.e., at ~35 to 90%; Fig. 3B). However, they were relatively ineffectual among those that were low in initial belief (i.e., climate skeptics). The main exception is in writing a letter to a member of the future-generation intervention, which worked across nearly the entire spectrum of initial belief. In addition, for those that were very low to moderate (i.e., 0 to 65%) on initial belief, the negative emotion intervention appeared to backfire, reducing support for climate change policies. Similar to belief, the work-together normative appeal also slightly backfired in participants with moderate levels of initial belief.

Regarding social media sharing, nearly all interventions (i.e., 9 of 11) increased willingness to share even at moderate levels of initial belief (i.e., those greater than ~35 to 60%). Moreover, the increase in willingness to share by inducing negative emotions extended into individuals who generally do not believe in climate change. Last, the work-together normative appeal intervention backfired among those who are very low on initial belief (i.e., ~0 to 15%), reducing their willingness to share information on social media by up to 12%. Last, for the tree-planting task, more than half of the interventions decreased the number of pages completed on the WEPT across all levels of initial belief (Fig. 3D).

### Country-level main effects

**Fig. 4.**
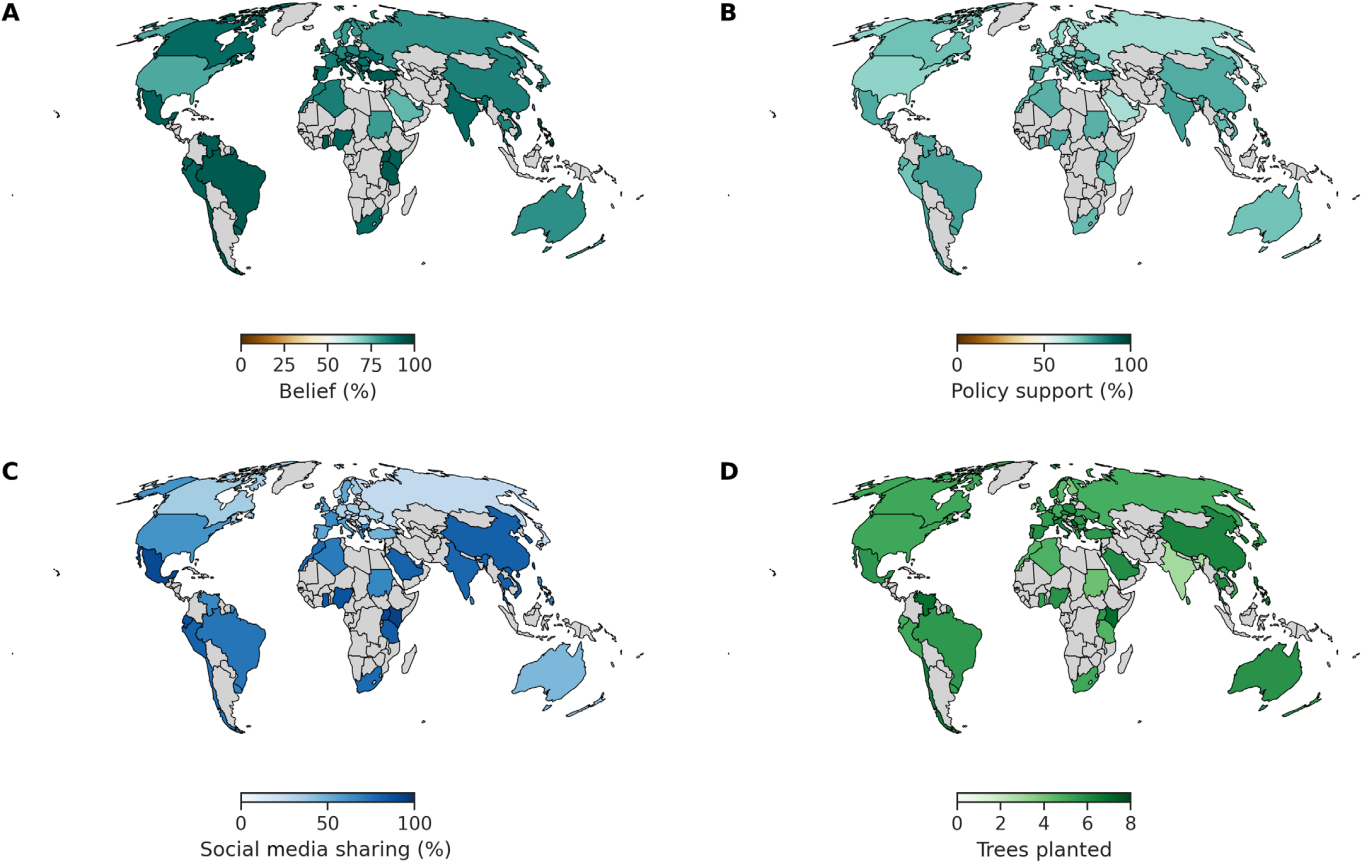
Country-level means of each outcome variable. Countries without available data are shown in gray. Statistics are shown in tables S5 to S8. (**A**) Climate change belief, (**B**) policy support, (**C**) sharing information on social media, and (**D**) trees planted via the WEPT.

Last, we examined the country-level main effects for each of our key outcome variables. We found that average belief in climate change, across all countries surveyed, was high [85.7% (85.2 to 86.2); this includes both ratings of belief in the control participants and estimated preintervention levels of belief from all other participants]. There was a very little variation between countries (Fig. 4A, fig. S4A, and table S5) indicating a clear majority belief in climate change. Similar patterns were observed for policy support (Fig. 4B), with all countries indicating clear majority support for a variety of climate change policies [72.2% (71.6 to 72.8)]. These results suggest that there is clear and consistent global consensus regarding the dangers posed by climate change and the importance of enacting climate change mitigation.

Other outcome variables exhibited larger variation across countries. Willingness to share climate change–related information on social media was more modest [56.9 (56.4 to 57.5)] and variable, ranging from low in Latvia of 17.6% (14.3 to 21.4) to high of 93.3% (90.4 to 95.7) in Kenya (Fig. 4C). These results suggest that observations of climate change discussion online may not accurately reflect global sentiments about the reality of climate change but rather different local norms. Last, half of all participants (50.7% of total sample and 53.1% of control condition sample) completed all eight pages of the WEPT, earning the maximum number of trees possible, with an overall average of 5.2 (5.1 to 5.3) pages completed (Fig. 4D).

## DISCUSSION

In a global megastudy conducted on a sample of 59,440 people from 63 countries, we empirically assessed the relative effectiveness of 11 expert-crowdsourced, theoretically derived behavioral interventions at stimulating climate mitigation beliefs and behaviors (i.e., climate change beliefs, policy support, willingness to share information, and tree-planting contributions). We found that different interventions tended to have small global effects, which varied across outcomes and largely affected nonskeptics, emphasizing the importance of examining the impact of climate interventions on a range of outcomes before drawing conclusions regarding their overarching relative efficacy. These findings suggest that the impact of behavioral climate interventions varies across audiences’ characteristics and target behaviors.

Here, climate change beliefs were strengthened most by decreasing the psychological distance of climate change. Support for climate change mitigation policy was increased mostly by writing a letter to be read in the future by a socially close child, describing one’s current climate change mitigation actions. Willingness to share climate change information on social media was increased most by inducing negative emotions through “doom and gloom”–styled messaging about the consequences of climate change. Last, while half of the tested interventions had no effect on the effortful tree-planting behavior, the other half of the interventions reduced the number of trees participants planted. Beyond revealing the utility of harnessing a multioutcome approach, these results also highlight the need for tailoring interventions to target outcomes.

Our findings extend prior work and are theoretically informative in several ways. Notably, these findings help reconcile several theoretical debates in the literature. For example, some have argued in favor of using a doom and gloom messaging style in climate communications (i.e., induce negative emotions) as a way to stimulate climate mitigation behaviors (*42*). For instance, recent work found that online news consumption is largely driven by the negative content of the news (*43*). However, others have warned that this messaging may have no impact on behavior (*44*) or, worse, that it may depress and demoralize the public into inaction (*45*). Here, we found empirical support for both accounts on different outcomes: While negative emotion messaging was highly effective at stimulating climate information–sharing intentions (a relatively low-effort behavior), it decreased tree-planting efforts. Further, the negative emotion induction intervention appeared to backfire on policy support among participants with low initial climate beliefs. These results suggest that climate scientists should carefully consider the differential effects of the prevalent fear-inducing writing styles on different proclimate outcomes. Moreover, it suggests that theoretical models need to explain divergent patterns across outcomes.

The results also indicate that the impact of the interventions on each outcome depends on peoples’ preexisting belief in climate change, supporting the claim that interventions need to be tailored to the characteristics of their audience (*44*, *45*). For belief, the effectiveness of several interventions (e.g., decreasing the psychological distance and collective action efficacy) was maximized among the uncertain, with lesser effects among believers and skeptics. For policy support, however, interventions were generally only effective among those with high initial levels of belief, with negative emotions backfiring among skeptics. Similarly, the robust increases in willingness to share on social media were largely restricted to people who already believed in climate change—with negative emotions increasing sharing intentions even among skeptics. For the higher effort behavior, however, interventions appeared to uniformly reduce tree planting across all levels of initial belief.

Given the heterogeneity of these results across outcomes, we created a web tool resource (https://climate-interventions.shinyapps.io/climate-interventions/) that can easily and rapidly assess intervention efficacy across each of the four outcomes and across a range of variables, including country, political ideology, gender, age, socioeconomic status, income, and education. While we caution that users must take into account the sample sizes when exploring subsamples of the data and the fact that they are looking at percentage of change compared to the control condition, this web tool can be used as a rapid and intuitive way to query intervention efficacy within subsamples of interest. For example, for highly educated conservatives in the United States, the top intervention to increase climate policy support was the future self-continuity intervention, increasing support by 18%. This intervention also increased climate beliefs in Russian participants by 9%. The scientific consensus intervention increased climate policy support by 9% in Romania but decreased it by 5% in Canada. The binding moral foundations intervention increased the number of trees planted by Australians under the age of 40 by 40%, and by Gambians by 35%, but this intervention decreased the number of trees planted by wealthy Japanese participants by 24%. These results can inform the development of local intervention strategies, which should then be empirically validated. Critically, these results also bolster the message that interventions need to be tailored to the characteristics of the target audience, nationality being an important factor. The accompanying data exploration web tool and the open-source raw dataset contribute to the data-as-public-good trend emerging in the spirit of open science, thus facilitating the testing of additional hypotheses and advancement of science.

In a linked forecasting experiment (*46*), academics (e.g., behavioral scientists) and the general public were asked to predict how each intervention would affect belief, policy support, and the tree-planting behavior in a subset of participants from this study (i.e., those from the United States). While academics were better than the general public at predicting the efficacy of these interventions on beliefs and policy support, when compared to statistical models using simple heuristics such as “interventions would have no effect,” no group was able to accurately predict how interventions would affect behavior. These results suggest that our findings here reflect an important departure from the expectations within the academic community.

There are also several limitations and future directions that should be emphasized. First, the sampling procedures differed between countries (e.g., the U.S. and Israel samples matched the census on age, gender, region, ethnicity; and the Norway sample matched on age, gender, ethnicity; etc.; Table 1). It should be noted that 73.6% of the entire sample was matched for at least one variable. However, despite these differences, recent work has found that representative samples are not required to obtain generalizable estimates of effect sizes within countries (*47*, *48*). Various analyses have highlighted that convenience samples are adequate for estimating treatment effects (*49*, *50*). Hence, given that our paper is primarily concerned with the effects of these interventions rather than with estimating levels of opinion within each country, our sampling procedures were appropriate for the analyses and conclusions drawn here. However, while realizing that it will be a challenge, we encourage future work to examine these processes using larger, more representative samples from an even broader sample of countries.

Second, we leveraged an online survey–based approach, which means that we were able to capture a limited set of contextual factors that may have influenced our results. This approach was the most effective way to measure and compare intervention efficacy in such a diverse global sample. However, one important and potentially impactful avenue for future research could be to leverage these findings to conduct local field experimentation in targeted samples.

One of the major strengths of our tournament was testing 11 different interventions simultaneously in a large global sample across multiple outcomes. Given the heterogeneity in the effectiveness of the interventions across the outcomes, future work should likewise prioritize testing promising interventions on even more climate-relevant antecedents and outcomes for a more comprehensive assessment of climate interventions and their underlying theoretical frameworks. One constraint we faced when attempting to test additional theories was the decision to not use deception in our interventions. For example, descriptive or injunctive norm–based interventions would have needed to be based on deception to be included in and deployed at this global scale, given the unavailability of the empirical information critical to creating these interventions. We hope that the current dataset can provide this information for future research in international contexts. Future work should also investigate additional proenvironmental behaviors, such as investment decisions, activism, advocacy, or civic participation, critical to climate change mitigation.

Future research should also assess the processes behind the negative effects we observed on the tree-planting task. Here, we find evidence for a trade-off between time spent on the intervention and in the behavioral task, but additional processes may also be at play. For instance, the negative effects observed might suggest a negative spillover process, by which increasing some mitigation actions (e.g., policy support, social media sharing, etc.) could have decreased other mitigation actions (e.g., contributing to tree planting). Given that the tree-planting task was also the last outcome variable completed by participants, such a process could be plausible. However, each of the first three outcomes (i.e., climate belief, climate policy support, and information-sharing willingness) was positively associated with the last outcome (i.e., WEPT; fig. S2 and tables S13 to S15). These positive associations at the study level also held within each of the 12 conditions (tables S16 to S18). That is, the more a participant supported climate policy, the more trees they planted, a pattern found under each condition (table S17). Similarly, participants who were willing to share climate information on social media also planted more trees, again a pattern found under each condition (table S18). These positive associations are more consistent with a positive spillover.

An alternative explanation for the intervention effects on the tree-planting task could be that current behavioral science theories and their corresponding interventions are more effective at targeting conceptual processes compared to more effortful and time-consuming behavioral signatures, especially in such a heterogeneous global sample. However, another explanation could be that interventions that made the negative consequences of climate change more salient (e.g., negative emotions, decreasing in psychological distance, and future self-continuity), triggered the perception that individual-level solutions (e.g., planting trees) may be futile in the face of such an insurmountable phenomenon, in line with the learned helplessness hypothesis (*45*). On the other hand, perhaps, a combination of these explanations gave rise to the effects observed. Future research is needed to clarify these processes and identify interventions that increase more effortful climate actions around the world, as well as actions that are more effective solutions to the climate crisis (*30*).

Last, while, in this global study, we tested the effects of several theoretically derived behavioral interventions on people’s beliefs and actions in the context of climate change, our findings provide meaningful insights to the broader fields of social and behavioral sciences. For instance, the average global effects of the interventions tested ranged from effectively zero to very small in the conceptual outcomes (beliefs and policy support) and near zero to negative in the behavioral outcome (tree planting). These findings point to critical limitations in these theories’ utility and generalizability beyond the contexts in which they were developed. The most extreme example is the correcting pluralistic ignorance intervention, which had no effect on beliefs, policy support, or willingness to share information on social media and even reduced tree-planting efforts. Theories are often tested and evaluated mainly on their ability to account for decontextualized patterns of data in laboratory settings, rather than their ability to help solve societal problems (*51*). In response to this limitation, researchers have recently proposed reverting the scientific paradigm to an impact-oriented theoretical and empirical research agenda (*30*).

The small effect sizes we observed in this global sample might also be partly interpreted through the lens of recent work reporting that over 60% of studies in the most prestigious journals in psychology have only focused on 11% of the world’s population (*52*). In our data collected in the United States or other WEIRD nations, the effects of the top interventions on belief and policy support were much stronger than at the global level. The skewed representation in the field may pose another notable obstacle in addressing societal problems that depend on global cooperation and a diversity of solutions for different cultural contexts, as is the case in climate change among numerous others global crises. One promising solution to these generalizability and practicality limitations in the behavioral sciences relies on embracing international collaborative science. Large global scientific projects can benefit from access to not only a wider range of populations but also from a diversity of scientific perspectives. For example, crowdsourcing has been found to improve the quality of scientific investigations by promoting ideation, inclusiveness, transparency, rigor, and reliability among other factors (*25*). Thus, crowdsourcing decisions related to the experimental design from experts more widely representative of the global scientific community might increase the impact and generalizability of scientific investigations. For example, the crowdsourcing of the theories tested from our large international team has led us to include less established interventions, such as “letter to future generation,” which ended up being one of the top interventions tested. Future work could also consider extending this crowdsourcing paradigm to include nonexperts (e.g., lay audiences), as recent work suggests that there may be unique benefits (e.g., increased interdisciplinarity), sometimes even producing research questions that outperform experts’ suggestions (*53*). Last, combining this “many labs” approach (*24*) with the megastudy approach (*18*) promises to push the limits of conventional scientific practices and overcome some of the main barriers of science generalization and implementation (*17*).

Overall, we tested the effectiveness of 11 expert-crowdsourced behavioral interventions at increasing climate awareness and action in 63 countries. Our findings provide theoretical support for many of the tested interventions. However, variation in effectiveness across outcomes, between countries, and along the spectrum of climate beliefs, suggests substantial gaps in our current theoretical understanding of climate change behavior. Moreover, the high preexisting levels of belief and policy support, alongside the small effect sizes observed here, raise critical questions about the practical capacity to facilitate bottom-up change at a global level, suggesting that top-down change might need to be prioritized to achieve the emissions reduction necessary to stay within safe planetary limits for human civilization. Practically, these findings provide critical information to policymakers considering climate solution implementations, streamlining the behavioral sciences’ response to the climate crisis.

## MATERIALS AND METHODS

### Participants

**Fig. 5.**
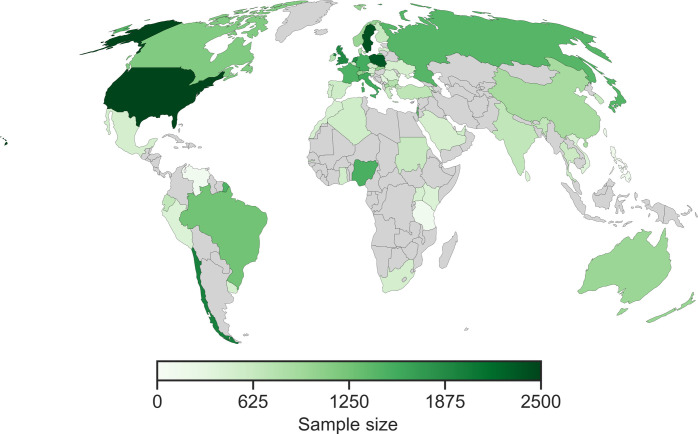
The number of participants in each of the 63 countries represented in the sample (*N*_total_ = 59,440).

The data were collected between July 2022 and July 2023 (see “note added in proof”). A total of 83,927 completed the study. Of them, 59,440 participants (*M*_age_ = 39.13, SD_age_ = 15.76; 50% women and 46% men) from 63 countries (Fig. 5 and Table 1) who passed the two attention checks (i.e., “Please select the color “purple” from the list below” and “To indicate you are reading this paragraph, please type the word sixty in the text box below”) and correctly completed the WEPT demo were included in the analyses. Although removing participants who failed these preregistered attention checks risks contributing to a selection bias in the sample (*54*), we a priori determined we would screen participants according to these criteria to ensure data quality.

Ethics approval was obtained independently by each data collection team from their corresponding Institutional Review Board. Only datasets submitted along with Institutional Review Board approval were included in the analysis.

### Collaboration procedure

Following procedures from Van Bavel and colleagues (*24*), the organizational team submitted a call for collaboration (https://manylabsclimate.wordpress.com/call-for-collaboration/) in November 2021 on social media (i.e., Twitter), via personal networks and by posting on various mailing lists. We asked researchers from around the world to join our project by contributing in one of three ways: (i) collecting data (i.e., >500 responses) from a country in which data had not already been collected, (ii) propose an intervention that becomes included in the final study, and/or (iii) fund data collection (i.e., >500 responses) from a country in which data had not already been collected and support a local team who lacks funding. The collaborators who proposed an intervention were asked to keep in mind time constraints (i.e., each intervention had to take on average at most 5 min) and the targeted outcome variables (i.e., climate beliefs, policy support, social media sharing, and tree-planting contributions). We received a total of 36 proposed interventions, which were coded by the first authors (who were blinded to the intervention authors). The coding procedure involved screening the proposed interventions for feasibility in an international context, relevance for the dependent variables, and theoretical support from prior work (quantified by previously reported effect sizes). We also aggregated similar interventions and duplicates. Following this procedure, we identified 11 unique and feasible interventions, which we then asked all collaborators to rate on perceived efficacy (practical support) and theoretical value (theoretical support), initially aiming to select the top five interventions. We obtained 188 responses from our collaborators in January 2022 (fig. S5). Given high support for all interventions, we decided to test all 11 interventions in the main study. We then contacted the collaborators whose interventions had been selected to be included in the main study, to coordinate the intervention implementation and programming on the Qualtrics survey platform (www.qualtrics.com/). After obtaining the programmed interventions, we gave our collaborators feedback on their submissions and allowed them time to address our comments. After receiving the revised interventions, we contacted expert researchers who had published theoretical work relevant to each intervention, asking them to critically review each intervention’s implementation. For example, Jost (*55*) reviewed the system justification intervention, and van der Linden *et al.* (*56*) reviewed the scientific consensus intervention. This process was iterated for each of the 11 interventions. After receiving critical suggestions from these experts, we engaged in another round of revisions. Last, in an attempt to reduce American-centric researcher biases, we asked all collaborators from around the world for additional feedback on the entire survey, including all interventions, demographics, and independent variables. This process lasted until the end of May 2022, when we started piloting the final version of the study, on a sample of 723 participants (*M*_age_ = 43.6; SD_age_ = 15.7; 52% women and 46% men, <2% nonbinary), collected in the United States. Using the pilot data, we wrote our analysis scripts and the preregistration (available at https://aspredicted.org/blind.php?x=W83_WTL). After the piloting was completed (July 2022), we sent our collaborators the final version of the study in Qualtrics along with an in-depth instructions manual (https://osf.io/ytf89/files/osfstorage/6454f8e3b30b49156cb9dd79/) on how to translate and adapt the study to each country. We also instructed our collaborators to obtain ethics approval from their International Review Boards before launching data collection. All collaborators were given 10 months (until May 2023) to submit their data.

### Experimental design

Participants signing up to complete the study (expected to take 15 min to complete) were first asked to read and sign the informed consent. They were then exposed to the first attention check (“Please select the color “purple” from the list below. We would like to make sure that you are reading these questions carefully.”), which removed from the experiment any participants choosing an incorrect answer. Then, participants were then given a definition of climate change: “Climate change is the phenomenon describing the fact that the world’s average temperature has been increasing over the past 150 years and will likely be increasing more in the future.” After reading this definition, participants were randomly assigned to 1 of 12 conditions: 11 experimental interventions (Fig. 1) or a no intervention control condition, in a between-subjects design. Participants under the control condition were then exposed to a short, thematically unrelated text from the novel *Great Expectations* by Charles Dickens, while participants under the experimental conditions were exposed to an intervention. Then, all participants were directed to the outcome variable phase, in which they rated (in random order) their (i) climate beliefs, (ii) climate policy support, and (iii) willingness to share climate information on social media. Last, participants were given the chance to contribute to tree-planting efforts by completing the WEPT. Then, participants under the control condition were asked to complete an additional set of variables. Last, all participants were asked to fill out a series of demographic variables, which included another attention check (“In the previous section, you viewed some information about climate change. To indicate you are reading this paragraph, please type the word sixty in the text box below.”)*.* Notably, participants filled out the entire survey in the primary language of their country of residence.

### Outcome variables

#### Climate beliefs

Climate beliefs were measured by participants’ answer to the question “How accurate do you think these statements are?” from 0 = not at all accurate to 100 = extremely accurate. The four statements were as follows: “Taking action to fight climate change is necessary to avoid a global catastrophe,” “Human activities are causing climate change,” “Climate change poses a serious threat to humanity,” and “Climate change is a global emergency.” The Cronbach’s alpha measure of internal consistency of this four-item scale in this dataset was 0.934.

#### Climate policy support

This dependent variable consisted of participants’ level of agreement from 0 = not at all to 100 = very much so, with the following nine statements: “I support raising carbon taxes on gas/fossil fuels/coal?,” “I support significantly expanding infrastructure for public transportation,” “I support increasing the number of charging stations for electric vehicles,” “I support increasing the use of sustainable energy such as wind and solar energy,” “I support increasing taxes on airline companies to offset carbon emissions,” “I support protecting forested and land areas,” “I support investing more in green jobs and businesses,” “I support introducing laws to keep waterways and oceans clean,” and “I support increasing taxes on carbon intense foods (for example meat and dairy).” The Cronbach’s alpha measure of internal consistency of this nine-item scale in this dataset was 0.876.

#### Social media sharing

Participants were first presented with the text, “Did you know that removing meat and dairy for only two of three meals per day could decrease food-related carbon emissions by 60%? It is an easy way to fight #ClimateChange #ManyLabsClimate${e://Field/cond} source: https://econ.st/3qjvOnn” (where “{e://Field/cond}” was replaced with the condition code for each group). Participants were then asked “Are you willing to share this information on your social media?,” the answer options being “Yes, I am willing to share this information,” “I am not willing to share this information,” and “I do not use social media.” Participants who indicated that they do not use social media were excluded from this analysis (i.e., a third of the sample). Moreover, participants were asked to indicate the platform (e.g., Facebook, Twitter, and Instagram) on which they posted the information.

#### WEPT tree-planting efforts

To measure an action with a real-world impact performed at an actual cost to participants, we used a modified version of the WEPT (*37*). This task is a multitrial web-based procedure that detects consequential proenvironmental behavior by allowing participants the opportunity of engaging in voluntary cognitive effort (i.e., screen numerical stimuli) in exchange for donations to an environmental organization. This measure has been validated and has been found to correlate with well-established scales for the assessing proenvironmental behavioral intentions (e.g., general ecological behavior scale) (*57*) and with direct donation behaviors (e.g., the donation of a part of their payment to an environmental organization) (*39*).

Participants were first exposed to a demonstration of the WEPT, in which they were instructed to identify all target numbers for which the first digit is even and the second digit is odd (4 of 18 numbers were target numbers on the demonstration page). Participants were not allowed to advance the page until they correctly completed the WEPT demonstration. Then, they were told that planting trees is one of the best ways to combat climate change and that they would have the opportunity to plant up to eight trees if they chose to engage in additional pages of the item identification task (one tree per page of WEPT completed). These pages contained 60 numbers per page, where participants had to screen for target numbers. Alongside these instructions, participants were shown a pictogram of eight trees, one of which was colored green to mark their progress in the task. Participants were allowed to exit the task at any point with no penalty.

#### Demographics

Participants were asked to indicate their gender, age, education level, political orientation for economic and social issues, and household income.

### Experimental conditions (interventions)

#### Working-together norms

The working-together norms intervention was submitted by M. Vlasceanu and J. Van Bavel. This intervention was adapted from Howe *et al.* (*11*), and it combines referencing a social norm with an invitation to work with others toward a common goal. This working-together normative appeal invites people to join in and “do it together” and has been found to increase interest in and actual charitable giving, reduce paper-towel use in public restrooms, and increase interest in reducing personal carbon emissions (*11*). Mediation analyses in prior work also suggested that working-together normative appeals are effective because they foster a feeling in participants that they are working together with others, which can increase motivation while reducing social pressure. Participants under this condition were exposed to a flier adapted from Howe and colleagues (*11*), after which they were asked 15 questions about the flier, serving as manipulation checks that were also meant to reinforce the manipulation [e.g., “If you are taking steps toward reducing your carbon footprint, to what extent would you feel like you are doing so together with other Americans (or participants’ group, adapted for each country)?” on a scale from 0 = not at all to 100 = extremely or “How strongly do you identify with your fellow Americans (or participants’ group, adapted for each country)?” on a scale from 0 = not at all to 100 = extremely].

#### System justification

SThe sstem justification intervention was submitted by O. Buchel, M. Tyrala, and A. Findor. This intervention is situated at the intersection of social identity, collective narcissism, and system justification approaches [based on (*58*)] and consists of framing climate change as uniquely threatening the way of life of participants’ nationality (e.g., the American way of life). Participants were asked to read a text emphasizing the importance of nature and the environment to one’s life [e.g., “(..) the food you eat, the sports you enjoy, the customs you observe, how you spend your free time, or even how you imagine growing old, all are likely impacted by where you live”], followed by examples of the effect of climate change on the local environment of participants’ nation [e.g., “(..) we can already see the consequences of climate change in the United States. For example, floods are becoming more and more frequent, putting a quarter of Americans at risk of losing their homes. Similarly, wildfires are becoming more frequent and more intense, threatening millions of Americans.”]. The text ends with an appeal to being proenvironmental as a patriotic gesture that will protect one’s way of life (e.g., “Being proenvironmental allows us to protect and preserve the American way of life. It is patriotic to conserve the country’s natural resources. It is important to protect and preserve our environment so that the United States remains the United States.”). This narrative was also intertwined with representative images of participants’ country of residence.

#### Binding moral foundations

The binding moral foundations intervention were submitted by B. Douglas and M. Brauer. This intervention relies on evoking ingroup-loyalty and authority moral foundations, which has been shown to increase support for proenvironmental behavior and attitudes (*59*, *60*). Participants were asked to read the following text “We are Americans (or participants’ nationality, adapted for each country). This means we can rise to any challenge that faces our country. From scientists to experts in the military, there is near universal agreement that climate change is real. The time to act is now. Using clean energy will help to keep our air, water, and land pure. It is the American (or participants’ nationality, adapted for each country) solution to the climate crisis,” after which they were exposed to an image of a person holding the national flag of participants’ country of residence.

#### Exposure to effective collective action

The exposure to effective collection action intervention was submitted by E. Shuman and A. Goldenberg. This intervention features examples of successful collective action that have had meaningful effects on climate policies, building on prior work showing that exposure to nonviolent action can increase willingness to join and maintain support (*61*, *62*). In addition, prior work also found that highlighting the possibility of making real concrete changes through collective action can increase hope, efficacy, and collective action (*61*). Participants were exposed to a text explaining that the impact people’s actions can have on curbing the effects of climate change, citing research indicating there is still “a window of opportunity” to make a difference. Then, participants were informed that the effectiveness of people’s actions to fight climate change depends on their ability to “come together and demand systemic change.” Participants were then exposed to several successful examples in which people solved global issues, such as the restoration of the ozone layer in 1987. Then, participants were exposed to examples of climate activism initialized by individual people and leading to large-scale movements or policy implementation (e.g., protests by locals from the American Midwest against fossil fuels pressured the governors of Illinois, Indiana, Michigan, Minnesota, and Wisconsin to build a new network for charging electric vehicles.). Images of concepts described in the text were displayed throughout.

#### Future self-continuity

The future self-continuity intervention was submitted by V. Ponizovskiy, L. Grigoryan, S. Grelle, and W. Hofmann. This intervention consists of emphasizing the future self that has been found in prior work to motivate future-oriented behaviors, such as academic performance, ethical decision making, and proenvironmental behavior (*63*–*65*). Participants were asked to read a text emphasizing the importance of engaging in climate action [i.e., “If no changes are made, the average temperature can increase by up to 6.5°C (12 F) by the year 2100 (IPCC, 2022). This would be extremely dangerous as super hurricanes, gigantic wildfires, and extreme food, and water shortages would become commonplace.”]. They were then presented with a series of causes for this phenomenon (i.e., “Human behaviors like energy production from fossil fuels, excessive meat consumption, and car driving increase the concentrations of greenhouse gasses in Earth’s atmosphere. Over 90% of the increase in the world’s temperature is caused by human activity.”). Last, participants were asked to imagine that their 2030 self is writing a letter to their present self, in which their future self is describing the actions they would have wanted to take regarding climate change [i.e., “Please put yourself in the year 2030—8 years from now. Take a few moments to imagine your life in that future. Imagine how you will look, where you will be, and who you are with. In the year 2030, it will be clear whether keeping climate change under 2°C is still possible. It will be clear whether the necessary change occurred fast enough to match the speed of the changing climate. As the Earth’s atmosphere continues to heat up, the effects of climate change will be more apparent: The “highest observed temperature” records will keep being updated, heatwaves and the draughts will become more common, species will continue to become extinct. Now please write yourself a “letter from the future.” This should be a letter you are writing in the year 2030, to your past self. As the person that you will be in 2030, what role would you think would be appropriate for you in respect to climate change? What would you want to tell yourself in the past? What would you like your past self to do? Please spend a bit of time on this task and try to write at least 100 words (five sentences), or more, if possible.”].

#### Scientific consensus

The scientific consensus intervention was submitted by A. van Stekelenburg, C. Klöckner, S. Vesely, and D. Bleize. This intervention consists of a message suggesting that climate scientists are in agreement with each other that climate change is real and primarily caused by human action. This messaging has been found to increase people’s belief in climate change and support for climate mitigation policy (*56*, *66*). Participants were exposed to the following text “Did you know that 99% of expert climate scientists agree that the Earth is warming and climate change is happening, mainly because of human activity (for example, burning fossil fuels)? [Myers *et al.* (*67*), Environmental Research Letters; Lynas *et al.* (*68*), Environmental Research Letters; Doran and Zimmerman (*69*), EOS]”. The text was accompanied by a pie chart with 99% of the surface area shaded.

#### Decreasing psychological distance

The decreasing psychological distance intervention was designed by S. Chamberlain, D. Hine, and G. Huang. This intervention is based on prior work finding that many perceive climate change as psychologically distant (i.e., “as a set of uncertain events that may occur far in the future, impacting distant places and affecting people dissimilar to themselves”) (*10*). Thus, framing climate change as a psychologically proximal risk issue (e.g., geographic) is expected to reduce psychological distance and increase public engagement. Participants were exposed to a paragraph emphasizing the impact of climate change (i.e., “There is no doubt that humans are the main driver of climate change. Human influence has warmed the atmosphere, ocean, and land. Climate change is already affecting every region across the world. It has resulted in more frequent and intense extreme weather events, causing widespread harm and damage to people, wildlife, and ecosystems. Human systems are being pushed beyond their ability to cope and adapt.”). They were then exposed to two examples of recent natural disasters caused by climate change in participants’ region (e.g., U.S. participants will be exposed to information about the 2021 record-breaking heat wave in North America causing the Lytton wildlife and to information about the 2017 Hurricane Harvey in Texas and Hurricane Irma in Florida, killing 232 people and causing $175 billion in damage). Participants were then asked to select the aspects of their lives affected by climate change from a list including: food production, farming and crop production, health and wellbeing, infectious disease, heat related harm and deaths, lack of, mental health issues, flooding and storms, changed land, freshwater and ocean environments, damaged infrastructure, and economy*.* After making the selections, participants were provided the correct answers based on current scientific estimates (i.e., all the possible options). Last, participants were asked to write about how climate change will affect them and their community (i.e., “Please write in a few sentences: How those climate consequences will affect you, your friends and family, and your community. Try to imagine these things happening today so you can be specific and describe what it will be like.”).

#### Dynamic social norms

The dynamic social norms intervention were submitted by O. Genschow, D. Loschelder, G. Sparkman, and K. C. Doell). This intervention is based on work showing that dynamic norms (i.e., how other people’s behavior is changing over time) are even more impactful at changing behavior than static social norms (*70*). Participants in this intervention first read a paragraph emphasizing that “People in the United States and around the world are changing: More and more people are concerned about climate change and are now taking action across multiple fronts,” accompanied by an image featuring relevant data in support of this claim. Then, participants were given examples of actions people are starting to take to mitigate the changing climate [i.e., “Since 2013, concerns about climate change have increased in most countries surveyed. What kinds of actions are people taking right now? More than ever before, people are making changes to their lifestyles, supporting policies to address climate change, and are giving the issue more time and attention. For example, more and more people from around the world are now cutting back on personal consumption, especially meat and dairy products, spending time, effort, and money on initiatives to mitigate climate change (for example, planting trees, offsetting carbon emissions), switching to low carbon modes of transportation (for example, taking bicycles). There’s also been a notable increase in support for climate change mitigation policy—some of the most popular policies include attempting to conserve forests and land, transitioning to solar, wind, and other renewable energy sources, creating/raising carbon taxes on fossil fuels, coal, gas, etc.”]*.*

#### Correcting pluralistic ignorance

The correcting pluralistic ignorance intervention was submitted by M. Schmitt, A. Lutz, and J. Lees. This intervention builds on work reporting that people substantially underestimate the climate change concern of others, a phenomenon labeled as “pluralistic ignorance” (*71*). Accordingly, collective action might be limited by people’s misperception that not many people are concerned. This intervention presented real public opinion data, which show that majorities around the world are concerned about climate change. Participants were first asked to predict the percent of people in their country who hold the belief that climate change is a global emergency [i.e., Researchers recently conducted the “People’s Climate Vote,” which is the World’s largest survey of public opinion on climate change (“global warming”). A total of 1.2 million people completed the survey from 50 different countries around the globe. The survey included people from the United States. Think for a moment about Americans and their views on climate change. How many Americans do you think would agree with the statement “Climate change is a global emergency”?]*.* After providing a prediction, participants were shown the actual percentage of people in their country who hold the belief in question, according to the Peoples’ Climate Vote (*72*). For example, participants in the United States will be told that “The People’s Climate Vote found that 65% of Americans agree that climate change is a global emergency”. For countries where the People’s Climate Vote does not report national level results, participants were presented with the climate opinion of people in their region.

#### Letter to future generations

The letter to future generations intervention was submitted by S. Syropoulos and E. Markowitz. This intervention involves writing a letter to a member of the future generation, which has been shown to reduce the psychological distance between one’s current choices and their consequences on future generations (*73*, *74*). Participants were asked to write a letter to a child who will read it in the future [i.e., “Please think of a child that is currently less than 5 years old (..) Now imagine that child is a 30-year-old adult. It is approximately the year 2055, they have started a family of their own, and they are finding their own way in the world. Whether they recognize it or not, they live in a world that is powerfully shaped by the decisions we are all making now, in 2022. One day, (..) they find a letter written today, in 2022, which is a message from you.”]. In this letter, participants are encouraged to write about their actions toward ensuring an inhabitable plant (i.e., “In it, you tell this family about all of the things you have done and want to do in the future to ensure that they will inherit a healthy, inhabitable planet. You tell them about your own personal efforts—however small or large—to confront the complex environmental problems of your time, from habitat loss to water pollution to climate change. In this letter, you also tell this family in 2055 about how you want to be remembered by them and future generations as someone who did their best to ensure a safe, flourishing world.”). Participants were allowed to write for 3 min and encouraged to write at least 100 words or 5 sentences.

#### Negative emotion

The negative emotion intervention was submitted by K. Doell and C. Pretus. This intervention involves exposure to scientific facts regarding the impacts of climate change in a doom and gloom messaging style typically used by climate communicators to induce negative emotions as a way of stimulating mitigation behaviors (*45*). Participants were first asked to report their baseline levels of emotions related to climate change, (e.g., hopeful, anxious, depressed, scared, indifferent, angry, helpless, and guilty). They were then exposed to information about the consequences of climate change alongside representative images [e.g., “Climate change is happening much more quickly and will have a much greater impact than climate scientists previously thought, according to the latest report by the Intergovernmental Panel on Climate Change (IPCC, 2022). If your anxiety about climate change is dominated by fears of starving polar bears, glaciers melting, and sea levels rising, you are barely scratching the surface of what terrors are possible, even within the lifetime of a young adult today. And yet the swelling seas—and the cities they will drown—have so dominated the picture of climate change/global warming that they have blinded us to other threats, many much closer at hand and much more catastrophic (...)”]. Last, participants were asked to report their levels of emotions related to climate change again.

### Control condition

Participants in the control condition were instructed to read a text retrieved from the novel *Great Expectations* by Charles Dickens [i.e., “As soon as the great black velvet pall outside my little window was shot with gray, I got up and went downstairs; every board upon the way and every crack in every board calling after me (…) I took it in the hope that it was not intended for early use and would not be missed for some time.”]. Participants were required to spend at least 10 s reading this text. This was to ensure that participants exerted some level of cognitive effort before being exposed to the dependent variable phase, to mirror the experience of participants in the experimental conditions. We chose a fiction text to prevent priming participants in any relevant way that could influence the dependent variables. After reading the excerpt, participants under the control condition were directed to the dependent variable phase, followed by the demographics phase. Last, participants under the control condition were also directed to an additional independent variable phase, exclusive to participants under this condition.

### Additional variables collected

These variables were only displayed to participants under the control condition, after they completed all dependent variables. First, participants were asked to rate the competence of climate scientists (“On average, how competent are climate change research scientists?” on a scale from 0 = not at all to 100 = very much so), their trust in scientific research about climate change (“On average, how much do you trust scientific research about climate change?” on a scale from 0 = not at all to 100 = very much so), their trust in their government (“On average, how much do you trust your government?” on a scale from 0 = not at all to 100 = very much so), their attitudes toward human welfare (“To what degree do you see yourself as someone who cares about human welfare?” on a scale from 0 = not at all to 100 = very much so), their global citizenship identity (“To what degree do you see yourself as a global citizen?” on a scale from 0 = not at all to 100 = very much so), their environmental identification (e.g., “To what degree do you see yourself as someone who cares about the natural environment?” on a scale from 0 = not at all to 100 = very much so), and their extrinsic environmental motivation (e.g., “Because of today’s politically correct standards, I try to appear proenvironmental.” on a scale from 0 = strongly disagree to 100 = strongly agree). Then, they were asked to estimate the percentage of people in their country who believe that climate change is a global emergency.

### Statistical methods

Our dependent variables have distributional properties (fig. S6) that preclude unbiased estimation with common, off-the-shelf, regression tools (such as the preregistered analyses). To address this, estimates presented in Fig. 2 relied on Bayesian methods and custom likelihood functions. Full mathematical descriptions of all models can be found in the supplied code (https://github.com/josephbb/ManyLabsClimate). Additional analyses can be found at https://github.com/mvlasceanu/ClimateTournament.

Belief was estimated using a hierarchical Zero-One-Inflated Beta (ZOIB) model. This model was further used to derive adjusted participant-level estimates of preintervention belief, to avoid postintervention bias in subsequent models. Sharing on social media was evaluated with a logistic regression. For WEPT, we used a geometric regression with a customized likelihood function to account for truncation and overinflation for the maximum number of trees planted. Priors were selected using prior-predictive simulation, with model structure iteratively developed through analysis of the prior predictive distribution and validated through model comparison using posterior predictive simulation. Posteriors were sampled using a No-U-Turn Sampler implemented on a graphics processing unit (GPU) with PyMC/NumPyro.

We note that these modeling choices are different from our preregistered analysis, which specified linear (belief and policy), ordinal (WEPT), and logistic (sharing) mixed-effects models. Plots of residuals from preregistered models suggested moderate to severe violations of distributional assumptions. For this reason, *P* values and estimates of effect sizes for these models may be unreliable. Despite these issues, we note that the findings from preregistered analyses are qualitatively similar to those from the Bayesian analyses. Overall, similarities between the preregistered and Bayesian analyses suggest effects that are remarkably robust to analysis decisions.

For completeness, we include the results as preregistered in tables S9 to S12 and fig. S1. Belief and policy support were modeled using a linear mixed-effects model with climate policy support as the dependent variable, condition as the fixed effect, including item (nine policies), participant, and country as random effects. WEPT was modeled using an ordinal mixed-effects model with climate action (WEPT) as the dependent variable and condition as the fixed effect, including country as random effects. Sharing was modeled using an ordinal mixed-effects model with climate action (WEPT) as the dependent variable and condition as the fixed effect, including country as random effects.

To develop and evaluate our Bayesian models, we adapted an established Principle Bayesian Workflow (*75*). This process begins by identifying inference goals, domain knowledge, and features of the dataset. Candidate statistical models are proposed, with prior predictive checks that are used to identify reasonable priors. Data are simulated from the prior predictive distribution, and the statistical model is fit to this simulated data. This allows for evaluation of computational properties of the model, tuning of the sampler, adjustment of the model or priors, and refinement. Key insight was gained through visual inspection of the posterior *z*-score versus posterior contraction, which can indicate issues with overfit, underfit, bad prior models, or poorly identified model specification. This process was iterated on until a suitable candidate model and priors were identified. Last, posterior predictive checks were used to verify that models adequately reconstructed broad properties of the data without regard to the estimands of interest (i.e., country/treatment effects). Failures here lead to adjustment of the underlying model. Once all model development criteria were satisfied, final analysis of the dataset was used to generate estimates of treatment and country level effects as well as all relevant figures. We note that priors for similar parameters across models may differ as a result of this iterative process, owing to distinct link functions and differing computational constraints. However, the impact of the prior on posterior samples is unlikely to be meaningful, given the volume of data.

We fit the selected model to the study data using PYMC (*76*) with a No U-Turn Sampler implemented on the GPU in NumPyro. We evaluated the model fit, ensuring the absence of divergent transitions, sufficient mixing of the (four) Markov chains, a large enough effective sample size, and an acceptable Estimated Bayesian Fraction of Missing Information. Last, data were simulated from the posterior distribution and visual inspection of these posterior retrodictive checks that were used to assess model fit. Sampling parameters were largely default and can be found in the supplied code.

#### Belief

Belief was indicated for four items on a scale from 0 to 100, inclusive. We scaled the outcome variable for each item to 0 to 1 to facilitate the use of common bound distributions. However, as both 0 and 1 were possible values, our likelihood function needed to account for possible inflation. Hence, we implemented a hierarchical ZOIB regression. We developed a generative model in which participants were estimated to have an unobserved preintervention belief, defined by their observed belief minus the estimated preintervention effect for their level of belief (i.e., as though they had been in the control condition) that was partially pooled by country, which, in turn, was partially pooled via a hyperparameter for average belief. Interventions were modeled with an intercept, corresponding to the average effect, and an effect of the estimated preintervention belief. The intervention effect and intercept for the control condition were fixed at zero. Otherwise, we modeled intervention effects using a multivariate normal distribution to account for covariance between intercepts and interventions. Further, we included partially pooled intercepts for item-specific effects. Where necessary, noncentered parameterizations were used to improve model fit.

Last, we extracted the posterior average preintervention belief for each participant to use in modeling policy support, social media sharing, and WEPT. This reflects the observed level of belief after adjusting for intervention effects on belief. As the treatment effects are small, these adjustments are minimal. Ideally, one would jointly model belief and other outcomes; however, the large sample sizes inherent to a megastudy impose computational constraints, a particular issue with model development and evaluation. Extracting intervention-adjusted estimates of initial belief enables us to examine heterogeneous intervention effects for each of these outcomes, at a tractable degree of model complexity. We chose to focus on belief for evaluating heterogeneous intervention effects under the assumption that belief is more likely to be a cause of support for policy, social media sharing, and investment in tree-planting activities than a consequence. Full mathematical descriptions of the model can be found in the supplied code.

#### Policy support

Support for policy was indicated for nine items on a scale from 0 to 100, inclusive. Because of computational constraints with the full dataset, we examined the average of these items. As with belief, this outcome was scaled from 0 to 1, and a ZOIB was used to model the data. Policy support was modeled with an intercept, an effect of adjusted belief, with intercept and belief effects modeled for interventions and countries. Intervention and country effects were modeled as separate zero-centered normal distributions.

#### Social media sharing

Sharing was a binary outcome, restricted to users who used social media. To analyze the impact on sharing, we relied on a Bayesian logistic regression. The probability of sharing was modeled with an intercept, an effect of adjusted belief, with intercept and belief effects modeled for interventions and countries. Intervention and country effects were modeled as separate zero-centered normal distributions.

#### Work for environmental protection task

Participants were able to plant between one and eight trees. We began by modeling this as a truncated geometric distribution, assuming that participants have a per–time step chance of giving up and are forced to stop at 8. However, we noticed an overabundance of planting eight trees consistent with some participants committing to planting all eight. Accordingly, we modified our likelihood to include inflation at eight trees. Posterior predictive fits confirmed adequate model fit. With this likelihood, we constructed a Bayesian hierarchical with an intercept, an effect of adjusted belief, and intercepts and belief effects modeled for interventions and countries.

*Note added in proof*: After this manuscript was accepted for publication, the authors alerted the editorial office to a paper they recently finalized that includes data used in this paper. This data can be found at: K. C. Doell, *et al*. The International Climate Psychology Collaboration: Climate change-related data collected from 63 countries. (2024). https://doi.org/10.31234/osf.io/7fy2g
