# Supplementary material for: Addressing climate change with behavioral science: A global intervention tournament in 63 countries
Source: Sci Adv. 2024 Feb 7;10(6):eadj5778. doi: 10.1126/sciadv.adj5778 (PMC10849597; doi:10.1126/sciadv.adj5778)
Supplement: Supplementary file 1 — Figs. S1 to S6 Tables S1 to S25 [file sciadv.adj5778_sm.pdf]

Science Advances
AAAS

Supplementary Materials for

Addressing climate change with behavioral science: A global intervention tournament in 63 countries

Madalina Vlasceanu *et al.*

Corresponding author: Madalina Vlasceanu, vlasceanu@nyu.edu; Kimberly C. Doell, kimberlycdoell@gmail.com

Sci. Adv. **10**, eadj5778 (2024)
DOI: 10.1126/sciadv.adj5778

This PDF file includes:

Figs. S1 to S6
Tables S1 to S25

Supplemental Figures and Tables

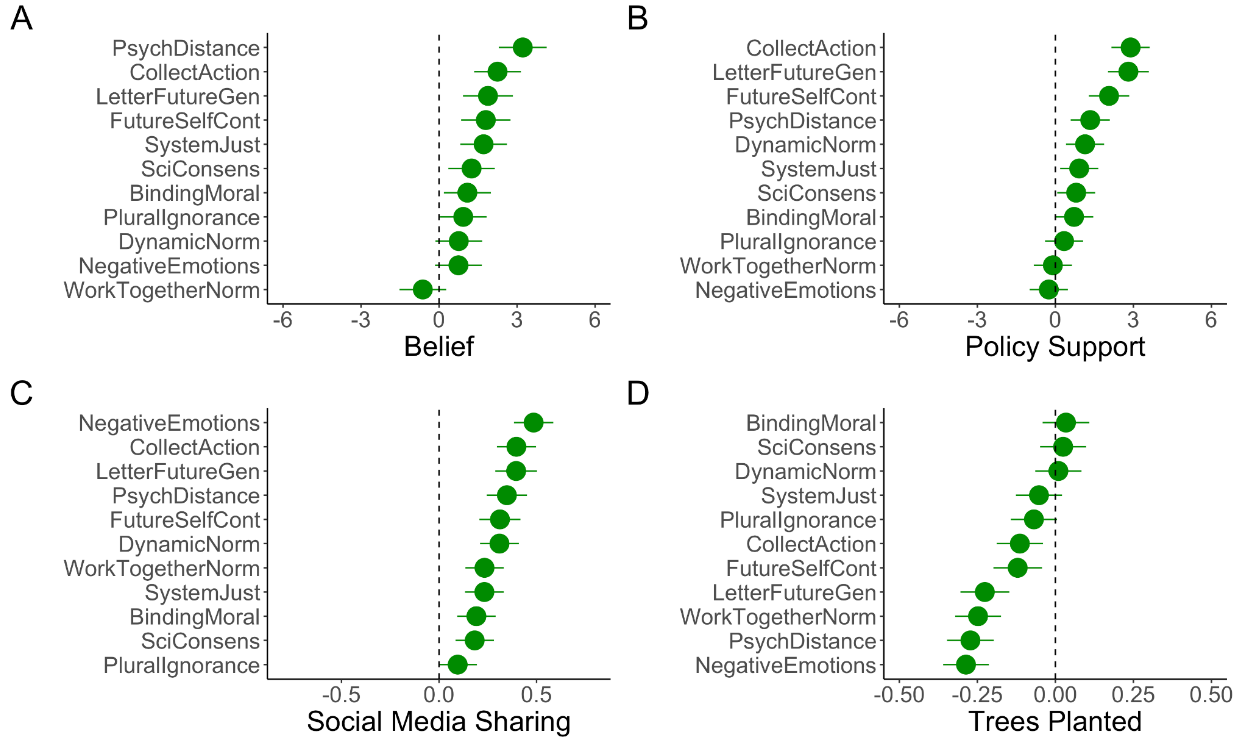

fig. S1: Coefficient estimates and 95% confidence intervals average treatment effects in the pre-registered analysis. A) Belief B) Policy Support C) Social Media Sharing D) Trees Planted (N=59,440 participants in 63 countries).

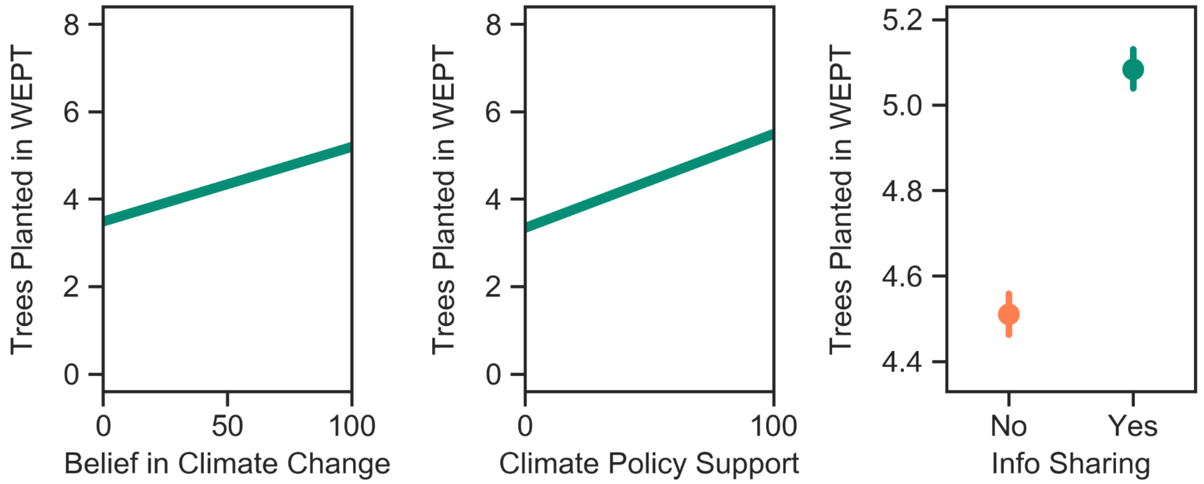

fig. S2: Number of trees planted in the WEPT as a function of belief in climate change, climate policy support, and willingness to share climate information. Statistics of the mixed models conducted are reported in tables S13-S18. The results reveal positive associations between the first three (lower effort behavioral) outcomes and the higher effort action (WEPT).

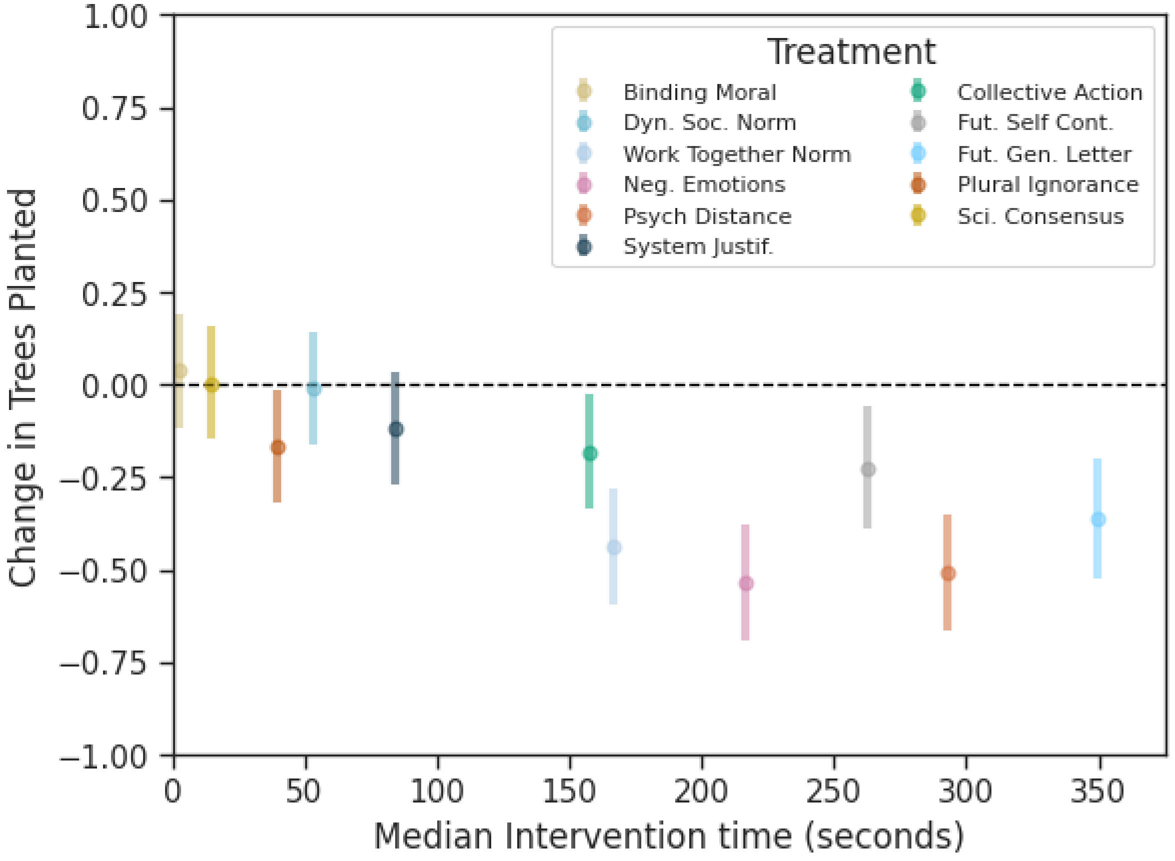

fig. S3: Average intervention effects for WEPT as a function of median intervention time.

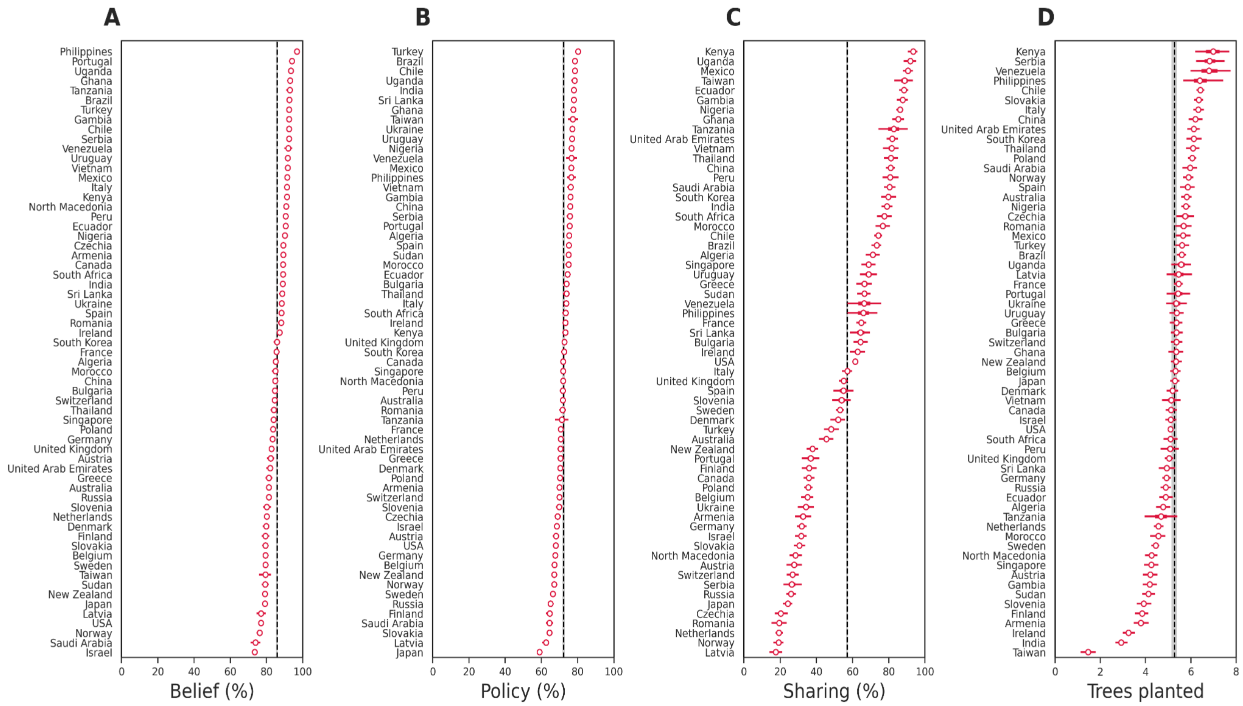

fig. S4: Country-level effects. Marginal, country-level posterior estimates for each of the key dependent variables. Dots indicate the mean, with error bars indicating the 94% credible region (C.R.). Thicker bars, when visible, indicate the interquartile range (IQR). Vertical lines and shading indicate the overall average across countries and 94% C.R., respectively. A) Belief, B) Support for Policy, C) Willingness to share climate-change information on social media, D) Number of trees planted in the WEPT task. Estimates shown in Tables S5-S8.

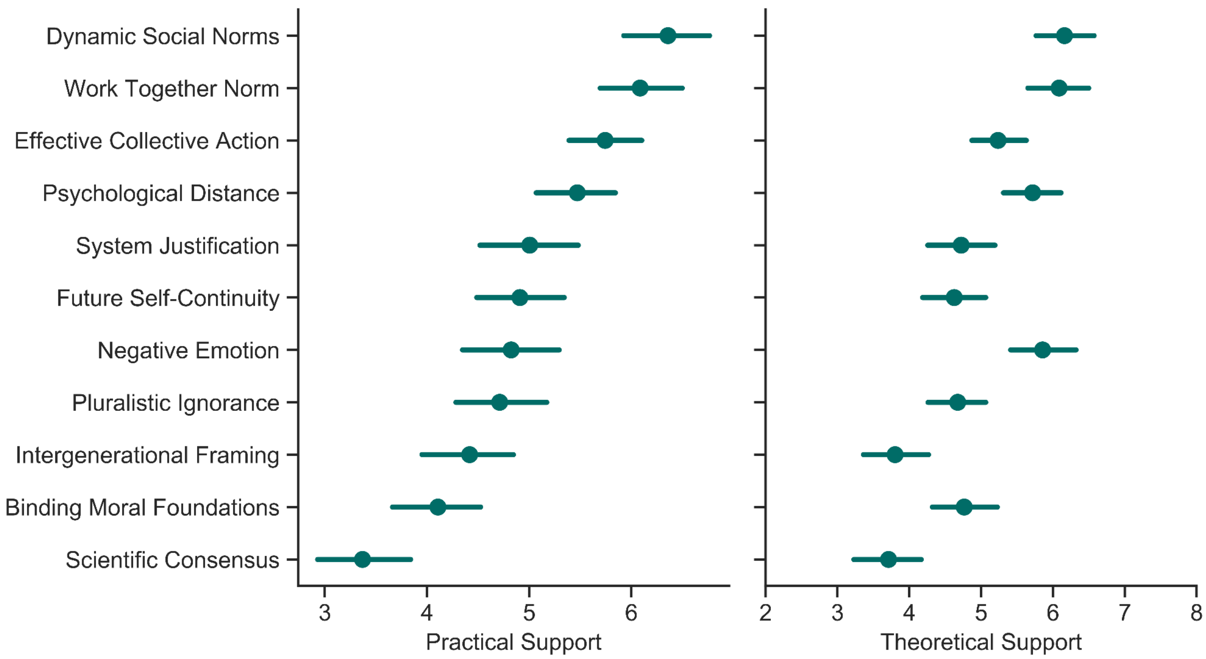

fig. S5. Average support of each crowdsourced intervention from a sample of 188 behavioral scientists (coauthors on the current paper) who were asked to rate the interventions on perceived efficiency (practical support) and theoretical value (theoretical support).

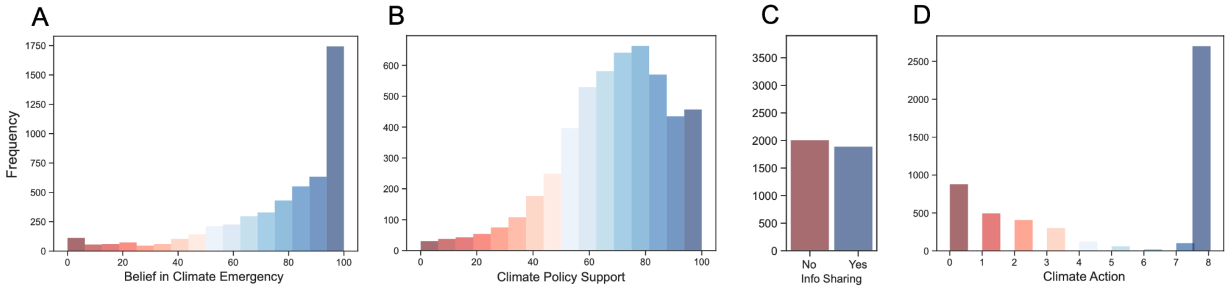

fig. S6. Frequency plots of A) belief, B) policy support, C) climate information sharing, and D) number of trees planted) in the control condition (N=5,086, from 63 countries), emphasizing the distributions of these dependent variables at baseline.

Table S1. Bayesian estimates of belief in climate change in each intervention, compared to the control condition.

Intervention	mean	sd	1.5%	3%	median	97%	98.5%
Psych Distance	2.255	0.332	1.524	1.619	2.263	2.865	2.954
Collective Action	1.454	0.338	0.712	0.816	1.457	2.07	2.177
Fut. Self Cont.	1.257	0.351	0.547	0.637	1.249	1.922	2.037
Letter Fut. Gen.	1.206	0.341	0.471	0.553	1.203	1.863	1.952
System Justif.	0.847	0.329	0.112	0.218	0.852	1.458	1.555
Sci. Consensus	0.426	0.345	-0.341	-0.252	0.437	1.052	1.134
Binding Moral	0.348	0.347	-0.385	-0.295	0.352	0.994	1.087
Dyn. Soc. Norm	0.343	0.342	-0.421	-0.324	0.35	0.969	1.073
Neg. Emotions	0.244	0.341	-0.488	-0.404	0.238	0.907	0.997
Plural Ignorance	-0.263	0.348	-0.976	-0.894	-0.274	0.404	0.507
Work Together Norm	-1.195	0.359	-1.947	-1.839	-1.198	-0.5	-0.374

Table S2. Bayesian estimates of policy support in each intervention, compared to the control condition.

Intervention	mean	sd	1.5%	3%	median	97%	98.5%
Letter Future Gen.	2.552	0.318	1.847	1.95	2.55	3.155	3.236
CollectAction	2.406	0.289	1.794	1.863	2.405	2.967	3.046
FutureSelfCont	2.111	0.308	1.464	1.545	2.111	2.696	2.804
PsychDistance	1.066	0.302	0.388	0.486	1.07	1.64	1.722
DynamicNorm	0.93	0.294	0.295	0.385	0.927	1.492	1.581
SystemJust	0.741	0.287	0.115	0.201	0.739	1.293	1.368
BindingMoral	0.511	0.298	-0.133	-0.051	0.506	1.062	1.159
SciConsens	0.471	0.309	-0.226	-0.098	0.468	1.041	1.135
NegativeEmotions	0.148	0.296	-0.501	-0.411	0.152	0.708	0.797
Work Together Norm	0.145	0.3	-0.485	-0.396	0.142	0.711	0.795
PluralIgnorance	0.096	0.299	-0.557	-0.463	0.094	0.666	0.737

Table S3. Bayesian estimates of sharing intentions in each intervention, compared to the control condition.

Intervention	mean	sd	1.5%	3%	median	97%	98.5%
Neg. Emotions	12.106	1.293	9.325	9.671	12.079	14.593	14.966
Letter Fut. Gen.	10.754	1.419	7.817	8.099	10.749	13.535	13.893
Collective Action	10.527	1.319	7.611	8.108	10.536	13.014	13.421
Psych Distance	9.085	1.338	6.288	6.631	9.074	11.611	11.974
Fut. Self Cont.	8.255	1.41	5.271	5.64	8.249	10.876	11.254
Dyn. Soc. Norm	7.978	1.317	5.085	5.465	7.982	10.455	10.847
Work Together Norm	7.535	1.331	4.739	5.111	7.515	10.078	10.395
System Justif.	5.812	1.304	2.966	3.367	5.801	8.301	8.639
Binding Moral	4.455	1.326	1.656	2.009	4.432	6.964	7.338
Sci. Consensus	4.444	1.352	1.527	1.851	4.449	6.964	7.388
Plural Ignorance	2.309	1.32	-0.51	-0.1	2.276	4.82	5.259

Table S4. Bayesian estimates of number of trees planted in each intervention, compared to the control condition.

Intervention	mean	sd	1.5%	3%	median	97%	98.5%
Binding Moral	0.038	0.082	-0.14	-0.117	0.039	0.192	0.213
Sci. Consensus	0.003	0.081	-0.17	-0.146	0.004	0.158	0.184
Dyn. Soc. Norm	-0.009	0.081	-0.182	-0.161	-0.008	0.144	0.171
System Justif.	-0.12	0.081	-0.295	-0.272	-0.12	0.031	0.057
Plural Ignorance	-0.165	0.081	-0.339	-0.321	-0.165	-0.014	0.008
Collective Action	-0.185	0.083	-0.361	-0.337	-0.187	-0.028	-0.005
Fut. Self Cont.	-0.229	0.086	-0.413	-0.391	-0.229	-0.061	-0.038
Letter Fut. Gen.	-0.365	0.087	-0.552	-0.526	-0.365	-0.199	-0.174
Work Together Norm	-0.438	0.08	-0.615	-0.593	-0.44	-0.283	-0.263
Psych Distance	-0.51	0.083	-0.689	-0.664	-0.509	-0.349	-0.326
Neg. Emotions	-0.536	0.082	-0.725	-0.695	-0.535	-0.377	-0.357

Table S5. Belief by country.

Country	mean	sd	1.5%	3%	median	97%	98.5%
Philippines	96.747	0.46	95.708	95.843	96.773	97.553	97.635
Portugal	94.055	0.451	93.031	93.157	94.072	94.89	94.99
Uganda	93.387	0.481	92.302	92.447	93.39	94.278	94.404
Ghana	92.957	0.489	91.872	92.014	92.963	93.867	93.989
Tanzania	92.854	1.084	90.229	90.67	92.915	94.733	94.991
Brazil	92.607	0.355	91.792	91.912	92.617	93.239	93.328
Turkey	92.478	0.447	91.522	91.629	92.477	93.293	93.402
Gambia	92.463	0.3	91.828	91.905	92.466	93.007	93.081
Chile	92.452	0.524	91.247	91.434	92.479	93.395	93.53
Serbia	92.398	0.642	90.878	91.134	92.411	93.626	93.816
Venezuela	91.975	1.179	89.143	89.564	92.049	94.006	94.291
Uruguay	91.697	0.578	90.342	90.559	91.723	92.707	92.883
Vietnam	91.631	0.676	90.067	90.339	91.654	92.847	93.026
Mexico	91.399	0.578	90.128	90.277	91.388	92.485	92.636
Italy	91.213	0.369	90.347	90.48	91.228	91.897	92.003
Kenya	91.167	0.698	89.535	89.759	91.208	92.367	92.525
North Macedonia	90.815	0.498	89.665	89.846	90.835	91.675	91.79
Peru	90.601	0.73	88.921	89.194	90.617	91.9	92.056
Ecuador	90.524	0.553	89.267	89.446	90.545	91.505	91.675
Nigeria	90.129	0.411	89.234	89.343	90.135	90.893	91.006
Czechia	89.293	0.7	87.628	87.889	89.314	90.554	90.792
Armenia	89.19	0.716	87.653	87.861	89.188	90.56	90.749
Canada	89.135	0.52	87.976	88.102	89.159	90.058	90.214
South Africa	89.102	0.767	87.409	87.612	89.127	90.537	90.681
India	88.96	0.664	87.432	87.654	88.98	90.168	90.389
Sri Lanka	88.652	0.865	86.66	86.929	88.682	90.198	90.408
Ukraine	88.368	0.759	86.643	86.881	88.406	89.702	89.894
Spain	88.212	0.781	86.348	86.653	88.233	89.699	89.897
Romania	88.046	0.883	86.064	86.301	88.058	89.647	89.865
Ireland	87.15	0.666	85.624	85.842	87.154	88.367	88.588
South Korea	85.666	0.812	83.785	84.024	85.699	87.144	87.398
France	85.492	0.592	84.076	84.292	85.506	86.551	86.707
Algeria	85.084	0.919	83.045	83.311	85.09	86.747	86.995
Morocco	84.872	0.971	82.576	82.881	84.926	86.581	86.805
China	84.692	0.709	83.101	83.32	84.711	85.955	86.16
Bulgaria	84.629	0.771	82.955	83.208	84.628	86.073	86.284
Switzerland	84.515	0.684	82.977	83.163	84.53	85.741	85.911
Thailand	83.984	0.967	81.835	82.115	84.022	85.761	85.979
Singapore	83.941	0.867	82.046	82.288	83.938	85.568	85.934
Poland	83.593	0.518	82.443	82.566	83.615	84.527	84.672

Germany	83.33	0.632	81.981	82.169	83.321	84.533	84.705
UK	82.663	0.608	81.265	81.516	82.674	83.764	83.941
Austria	82.254	1.04	79.734	80.151	82.305	84.075	84.398
UAE	81.942	1.016	79.602	79.966	81.958	83.751	84.045
Greece	81.318	1.051	78.973	79.264	81.332	83.301	83.531
Australia	81.316	0.82	79.469	79.7	81.345	82.823	83.037
Russia	81.226	0.708	79.681	79.887	81.235	82.584	82.728
Slovenia	80.135	1.18	77.379	77.743	80.189	82.23	82.445
Netherlands	80.092	0.677	78.589	78.784	80.099	81.354	81.533
Denmark	79.843	0.972	77.656	77.993	79.844	81.63	81.926
Finland	79.436	1.127	77.014	77.333	79.425	81.454	81.737
Slovakia	79.396	0.89	77.342	77.609	79.438	80.904	81.136
Belgium	79.384	0.875	77.393	77.659	79.424	80.966	81.165
Sweden	79.343	0.648	77.842	78.062	79.333	80.527	80.699
Taiwan	79.281	1.756	75.177	75.899	79.342	82.588	83.03
Sudan	79.217	1.058	76.913	77.229	79.241	81.058	81.214
New Zealand	79.152	0.859	77.212	77.488	79.169	80.748	80.977
Japan	79.148	0.733	77.501	77.759	79.147	80.443	80.629
Latvia	76.918	1.378	73.982	74.384	76.929	79.475	79.801
USA	76.861	0.467	75.819	75.945	76.882	77.687	77.8
Norway	76.248	0.898	74.234	74.541	76.256	77.931	78.188
Saudi Arabia	73.952	1.469	70.969	71.339	73.941	76.716	77.138
Israel	73.439	0.891	71.426	71.688	73.468	75.037	75.294

Table S6. Policy support by country.

Country	mean	sd	1.5%	3%	median	97%	98.5%
Turkey	80.201	0.635	78.839	79.014	80.185	81.407	81.573
Brazil	78.445	0.565	77.204	77.35	78.442	79.503	79.643
Chile	78.425	0.469	77.42	77.547	78.429	79.31	79.438
Uganda	78.278	0.893	76.328	76.582	78.272	79.967	80.232
India	77.921	0.647	76.479	76.687	77.926	79.164	79.342
Sri Lanka	77.858	0.767	76.13	76.388	77.849	79.3	79.52
Ghana	77.564	0.744	75.985	76.178	77.554	78.958	79.201
Taiwan	77.336	1.48	74.05	74.497	77.335	80.053	80.405
Ukraine	76.983	0.724	75.392	75.62	76.997	78.318	78.554
Uruguay	76.859	0.763	75.198	75.402	76.867	78.313	78.496
Nigeria	76.617	0.512	75.497	75.653	76.619	77.569	77.714
Venezuela	76.565	1.584	73.061	73.495	76.589	79.495	79.863
Mexico	76.361	0.827	74.582	74.852	76.365	77.92	78.152
Philippines	76.353	1.293	73.538	73.903	76.38	78.786	79.061
Vietnam	76.083	0.932	73.989	74.3	76.096	77.784	78.084
Gambia	75.981	0.793	74.187	74.467	75.989	77.488	77.73
China	75.82	0.649	74.416	74.624	75.808	77.069	77.271
Serbia	75.646	0.934	73.624	73.868	75.656	77.348	77.657
Portugal	75.578	0.809	73.839	74.051	75.566	77.121	77.359
Algeria	75.326	0.759	73.669	73.888	75.319	76.722	76.976
Spain	75.11	0.838	73.305	73.558	75.125	76.663	76.945
Sudan	75.01	0.809	73.256	73.441	75.021	76.491	76.666
Morocco	74.933	0.892	73.007	73.267	74.925	76.637	76.907
Ecuador	74.365	0.76	72.765	72.986	74.358	75.799	75.993
Bulgaria	73.86	0.764	72.147	72.436	73.864	75.284	75.522
Thailand	73.766	0.932	71.752	71.954	73.781	75.476	75.78
Italy	73.757	0.543	72.576	72.763	73.755	74.782	74.927
South Africa	73.328	0.895	71.409	71.653	73.328	75.024	75.341
Ireland	73.256	0.735	71.7	71.859	73.255	74.615	74.779
Kenya	73.196	0.885	71.283	71.53	73.205	74.859	75.06
UK	72.706	0.61	71.391	71.559	72.71	73.877	74.085
South Korea	72.512	0.829	70.713	70.914	72.52	74.064	74.302
Canada	72.018	0.661	70.594	70.774	72.007	73.262	73.445
Singapore	71.941	0.887	70.005	70.292	71.936	73.608	73.864
North Macedonia	71.941	0.652	70.468	70.75	71.963	73.132	73.365
Peru	71.87	0.93	69.824	70.114	71.865	73.592	73.83
Australia	71.744	0.748	70.123	70.312	71.731	73.187	73.399
Romania	71.679	0.983	69.539	69.809	71.701	73.531	73.792
Tanzania	71.283	1.955	66.959	67.596	71.282	74.866	75.397
France	70.641	0.612	69.278	69.475	70.646	71.785	71.944

Netherlands	70.631	0.586	69.356	69.554	70.639	71.729	71.892
UAE	70.519	0.928	68.478	68.759	70.536	72.248	72.512
Greece	70.466	0.803	68.725	68.978	70.471	71.986	72.194
Denmark	70.356	0.84	68.5	68.761	70.359	71.915	72.177
Poland	70.223	0.509	69.155	69.301	70.212	71.199	71.354
Armenia	69.95	0.812	68.195	68.479	69.932	71.495	71.768
Switzerland	69.881	0.725	68.28	68.481	69.878	71.207	71.431
Slovenia	69.713	0.896	67.822	68.03	69.709	71.384	71.599
Czechia	68.975	0.906	66.989	67.269	68.965	70.681	70.886
Israel	68.435	0.591	67.119	67.298	68.437	69.543	69.756
Austria	68.046	0.949	66.023	66.281	68.058	69.777	70.017
USA	67.88	0.431	66.916	67.036	67.887	68.671	68.771
Germany	67.674	0.643	66.299	66.474	67.67	68.908	69.099
Belgium	67.118	0.746	65.512	65.722	67.098	68.528	68.735
New Zealand	67.028	0.767	65.367	65.599	67.027	68.481	68.697
Norway	67.013	0.758	65.327	65.539	67.009	68.436	68.66
Sweden	66.273	0.58	64.976	65.172	66.268	67.352	67.507
Russia	64.992	0.579	63.756	63.92	64.986	66.103	66.259
Finland	64.545	0.994	62.353	62.61	64.544	66.362	66.646
Saudi Arabia	64.444	1.093	62.171	62.508	64.416	66.544	66.834
Slovakia	64.362	0.703	62.865	63.038	64.358	65.689	65.858
Latvia	62.491	0.983	60.326	60.591	62.512	64.358	64.592
Japan	58.939	0.616	57.613	57.798	58.945	60.097	60.275

Table S7. Sharing intentions by country.

Country	mean	sd	1.5%	3%	median	97%	98.5%
Kenya	93.31	1.437	89.913	90.418	93.426	95.709	95.969
Uganda	91.743	1.88	87.214	87.855	91.873	94.834	95.339
Mexico	90.518	1.53	86.866	87.42	90.596	93.149	93.489
Taiwan	88.544	2.757	81.7	83.04	88.705	93.337	94.025
Ecuador	88.295	1.408	85.068	85.514	88.361	90.728	91.051
Gambia	87.53	1.656	83.911	84.365	87.581	90.524	91.035
Nigeria	86.151	0.977	83.916	84.251	86.166	87.89	88.244
Ghana	85.086	1.758	81.183	81.675	85.133	88.257	88.665
Tanzania	82.466	4.43	72.229	73.671	82.664	90.198	90.939
UAE	81.872	1.717	78.087	78.663	81.876	84.983	85.388
Vietnam	81.416	2.309	76.107	76.954	81.581	85.437	86.051
Thailand	81.093	2.097	76.417	77.093	81.121	84.968	85.517
China	80.925	1.44	77.75	78.191	80.932	83.51	83.837
Peru	80.692	2.314	75.356	76.058	80.749	84.931	85.465
Saudi Arabia	80.387	1.778	76.361	76.968	80.417	83.59	84.102
South Korea	79.702	2.197	74.722	75.38	79.756	83.727	84.351
India	78.964	1.608	75.443	75.878	78.991	81.939	82.332
South Africa	77.534	2.145	72.813	73.381	77.549	81.466	82.084
Morocco	76.654	2.116	71.898	72.58	76.713	80.525	81.113
Chile	74.184	1.191	71.658	71.97	74.167	76.464	76.76
Brazil	73.233	1.409	70.123	70.521	73.256	75.824	76.273
Algeria	71.205	2.15	66.353	67.162	71.19	75.038	75.525
Singapore	68.836	2.176	64.203	64.819	68.883	72.738	73.444
Uruguay	68.78	2.526	63.148	63.917	68.883	73.361	74.062
Greece	66.537	2.305	61.533	62.161	66.559	70.846	71.454
Sudan	66.491	1.941	62.435	62.844	66.5	70.158	70.738
Venezuela	66.344	4.934	55.454	56.782	66.413	75.446	77.046
Philippines	65.902	4.414	56.219	57.384	65.891	73.895	75.357
France	64.752	1.5	61.54	61.94	64.755	67.583	67.945
Sri Lanka	64.328	2.918	57.922	58.69	64.353	69.722	70.551
Bulgaria	64.26	2.122	59.538	60.124	64.339	68.201	68.753
Ireland	62.759	2.204	57.926	58.503	62.775	66.783	67.301
USA	61.391	0.682	59.916	60.119	61.385	62.695	62.902
Italy	57.09	1.491	53.891	54.306	57.102	59.856	60.285
UK	55.063	1.393	52.023	52.497	55.092	57.584	57.986
Spain	54.985	3.029	48.591	49.382	54.984	60.563	61.501
Slovenia	53.929	2.686	48.062	48.817	53.924	59.043	59.99
Sweden	53.058	1.234	50.478	50.752	53.076	55.32	55.776
Denmark	51.981	2.146	47.428	47.898	51.99	56.016	56.542
Turkey	48.186	2.195	43.447	44.117	48.156	52.302	52.844

Australia	45.53	2.075	40.871	41.597	45.541	49.522	50.215
New Zealand	37.913	1.782	33.92	34.598	37.917	41.228	41.754
Portugal	36.846	2.624	31.254	32.018	36.884	41.748	42.542
Finland	35.997	2.217	31.283	31.857	35.971	40.224	40.93
Canada	35.824	1.684	32.27	32.678	35.782	38.918	39.367
Poland	35.555	1.239	32.906	33.248	35.562	37.891	38.317
Belgium	35.172	1.781	31.455	31.861	35.137	38.561	39.07
Ukraine	34.285	2.412	29.372	29.887	34.258	38.955	39.714
Armenia	32.677	2.393	27.546	28.264	32.635	37.313	37.996
Germany	31.981	1.466	28.809	29.274	31.954	34.732	35.24
Israel	31.629	1.68	28.09	28.442	31.617	34.834	35.395
Slovakia	30.655	1.647	27.065	27.637	30.641	33.806	34.307
North Macedonia	28.421	1.871	24.443	24.903	28.461	31.937	32.364
Austria	27.791	2.325	22.971	23.578	27.753	32.242	32.957
Switzerland	26.868	1.743	23.171	23.71	26.854	30.263	30.85
Serbia	26.537	2.713	21.029	21.639	26.433	31.747	32.44
Russia	26.052	1.41	23.027	23.445	26.03	28.729	29.216
Japan	24.205	1.415	21.274	21.618	24.199	26.888	27.234
Czechia	20.411	1.909	16.319	16.887	20.369	24.17	24.672
Romania	19.597	2.24	15.141	15.559	19.52	24.07	24.782
Netherlands	19.335	1.113	17.006	17.313	19.325	21.478	21.761
Norway	19.178	1.467	16.118	16.46	19.168	22.081	22.577
Latvia	17.684	1.9	13.816	14.27	17.629	21.378	22.103

Table S8. Number of trees planted by country.

Country	mean	sd	1.5%	3%	median	97%	98.5%
Kenya	6.958	0.42	6.048	6.167	6.978	7.678	7.745
Serbia	6.851	0.346	6.188	6.268	6.824	7.55	7.636
Venezuela	6.815	0.485	5.819	5.939	6.796	7.734	7.811
Philippines	6.447	0.459	5.606	5.712	6.393	7.52	7.676
Chile	6.418	0.09	6.222	6.245	6.418	6.583	6.613
Slovakia	6.348	0.108	6.098	6.14	6.349	6.549	6.585
Italy	6.33	0.129	6.062	6.101	6.327	6.582	6.616
China	6.204	0.161	5.878	5.917	6.198	6.525	6.587
UAE	6.126	0.145	5.817	5.853	6.126	6.4	6.435
South Korea	6.126	0.177	5.761	5.808	6.122	6.481	6.55
Thailand	6.085	0.16	5.744	5.789	6.088	6.385	6.438
Poland	6.057	0.098	5.853	5.878	6.057	6.239	6.27
Saudi Arabia	5.959	0.173	5.584	5.635	5.96	6.288	6.344
Norway	5.894	0.121	5.629	5.662	5.895	6.125	6.15
Spain	5.855	0.173	5.486	5.529	5.855	6.184	6.24
Australia	5.807	0.12	5.53	5.576	5.81	6.023	6.054
Nigeria	5.776	0.106	5.545	5.575	5.776	5.972	6.002
Czechia	5.75	0.209	5.329	5.372	5.745	6.167	6.23
Romania	5.674	0.194	5.263	5.322	5.67	6.046	6.1
Mexico	5.659	0.17	5.296	5.343	5.657	5.981	6.03
Turkey	5.607	0.161	5.266	5.312	5.602	5.918	5.965
Brazil	5.591	0.114	5.339	5.375	5.591	5.798	5.832
Uganda	5.576	0.239	5.116	5.164	5.565	6.052	6.154
Latvia	5.47	0.308	4.884	4.948	5.45	6.08	6.183
France	5.449	0.104	5.221	5.25	5.451	5.639	5.668
Portugal	5.446	0.28	4.913	4.979	5.427	6.023	6.137
Ukraine	5.369	0.247	4.878	4.938	5.356	5.874	5.977
Uruguay	5.369	0.172	5.005	5.047	5.368	5.691	5.743
Greece	5.368	0.152	5.041	5.084	5.367	5.649	5.696
Bulgaria	5.365	0.141	5.065	5.102	5.362	5.634	5.68
Switzerland	5.359	0.132	5.067	5.112	5.356	5.615	5.658
Ghana	5.348	0.171	4.976	5.022	5.35	5.679	5.735
New Zealand	5.34	0.129	5.062	5.096	5.341	5.58	5.613
Belgium	5.317	0.123	5.05	5.083	5.316	5.555	5.586
Japan	5.296	0.108	5.059	5.094	5.295	5.5	5.523
Denmark	5.189	0.134	4.897	4.938	5.19	5.442	5.483
Vietnam	5.137	0.222	4.669	4.729	5.131	5.561	5.619
Canada	5.121	0.132	4.832	4.87	5.118	5.372	5.414
Israel	5.102	0.127	4.831	4.867	5.103	5.337	5.376
USA	5.097	0.065	4.953	4.978	5.096	5.22	5.239

South Africa	5.096	0.169	4.726	4.77	5.101	5.411	5.449
Peru	5.083	0.212	4.623	4.691	5.082	5.493	5.557
UK	5.034	0.103	4.813	4.839	5.035	5.225	5.253
Sri Lanka	4.928	0.19	4.519	4.566	4.931	5.282	5.334
Germany	4.919	0.104	4.694	4.725	4.918	5.115	5.139
Russia	4.893	0.119	4.635	4.669	4.892	5.119	5.16
Ecuador	4.887	0.163	4.541	4.585	4.885	5.197	5.24
Algeria	4.772	0.167	4.417	4.456	4.773	5.086	5.134
Tanzania	4.68	0.387	3.862	3.969	4.675	5.421	5.561
Netherlands	4.567	0.107	4.332	4.363	4.565	4.776	4.806
Morocco	4.557	0.178	4.177	4.224	4.558	4.896	4.951
Sweden	4.441	0.093	4.233	4.265	4.442	4.609	4.641
North Macedonia	4.262	0.146	3.961	3.991	4.259	4.539	4.584
Singapore	4.25	0.172	3.875	3.929	4.25	4.567	4.616
Austria	4.204	0.172	3.828	3.88	4.201	4.536	4.575
Gambia	4.179	0.173	3.806	3.852	4.181	4.503	4.562
Sudan	4.128	0.152	3.804	3.843	4.126	4.418	4.462
Slovenia	3.918	0.175	3.536	3.593	3.915	4.251	4.299
Finland	3.843	0.156	3.507	3.545	3.844	4.133	4.177
Armenia	3.792	0.176	3.419	3.466	3.79	4.13	4.174
Ireland	3.246	0.145	2.932	2.979	3.24	3.526	3.561
India	2.921	0.143	2.631	2.667	2.918	3.199	3.233
Taiwan	1.469	0.184	1.107	1.153	1.459	1.836	1.908

Table S9: Coefficient table from pre-registered analysis of climate beliefs. Results are from a linear mixed effects model with climate beliefs as the dependent variable, condition as the fixed effect, including item (4 beliefs), participant, and country as random effects. Estimates are shown relative to the Control Condition.

Intervention	Estimate	SE	*df*	*t*	*d*	*p*
(Intercept)	79.98	0.88	79.30	91.20	20.48	**< .001**
PsychDistance	3.22	0.47	59166.56	6.86	0.06	**< .001**
CollectAction	2.25	0.46	59166.10	4.90	0.04	**< .001**
LetterFutureGen	1.88	0.49	59170.10	3.84	0.03	**< .001**
SystemJust	1.71	0.46	59170.14	3.74	0.03	**< .001**
FutureSelfCont	1.80	0.48	59176.06	3.72	0.03	**< .001**
SciConsens	1.25	0.46	59168.87	2.75	0.02	**0.006**
BindingMoral	1.09	0.46	59172.21	2.37	0.02	**0.018**
PluralIgnorance	0.93	0.46	59161.92	2.03	0.02	**0.042**
DynamicNorm	0.76	0.46	59185.00	1.66	0.01	0.098
NegativeEmotions	0.75	0.46	59166.90	1.63	0.01	0.103
WorkTogetherNorm	-0.62	0.46	59160.72	-1.36	-0.01	0.174

Table S10: Coefficient table from pre-registered analysis of climate policy support. Results are from a linear mixed effects model with climate policy support, as the dependent variable, condition as the fixed effect, including item (9 policies), participant, and country as random effects. Estimates are shown relative to the Control Condition.

Intervention	Estimate	SE	*df*	*t*	*d*	*p*
(Intercept)	70.23	4.02	8.42	17.45	12.03	**< .001**
CollectAction	2.89	0.38	58566.33	7.71	0.06	**< .001**
LetterFutureGen	2.81	0.40	58603.94	7.01	0.06	**< .001**
FutureSelfCont	2.06	0.40	58598.68	5.21	0.04	**< .001**
PsychDistance	1.34	0.38	58643.56	3.49	0.03	**< .001**
DynamicNorm	1.14	0.38	58602.04	3.04	0.03	**0.002**
SystemJust	0.91	0.37	58568.86	2.44	0.02	**0.015**
SciConsens	0.80	0.37	58609.08	2.14	0.02	**0.032**
BindingMoral	0.72	0.38	58582.48	1.91	0.02	0.057
PluralIgnorance	0.33	0.37	58578.83	0.89	0.01	0.373
WorkTogetherNorm	-0.10	0.38	58621.02	-0.26	0.00	0.794
NegativeEmotions	-0.25	0.38	58576.15	-0.68	-0.01	0.499

Table S11: Coefficient table from pre-registered analysis of Social Media Sharing. Results are from logistic mixed effects model with social media sharing as the dependent variable, condition as the fixed effect, including country as random effects. Estimates are shown relative to Control.

Intervention	Estimate	SE	*z*	*d*	*p*
(Intercept)	0.08	0.15	0.51	0.04	0.611
NegativeEmotions	0.49	0.05	9.43	0.27	**< .001**
CollectAction	0.40	0.05	7.76	0.22	**< .001**
LetterFutureGen	0.40	0.06	7.24	0.22	**< .001**
PsychDistance	0.35	0.05	6.64	0.19	**< .001**
DynamicNorm	0.31	0.05	6.12	0.17	**< .001**
FutureSelfCont	0.31	0.05	5.82	0.17	**< .001**
WorkTogetherNorm	0.23	0.05	4.65	0.13	**< .001**
SystemJust	0.23	0.05	4.60	0.13	**< .001**
BindingMoral	0.19	0.05	3.81	0.11	**< .001**
SciConsens	0.18	0.05	3.65	0.10	**< .001**
PluralIgnorance	0.10	0.05	1.90	0.05	0.057

Table S12: Coefficient table from pre-registered analysis of WEPT. Results are from an ordinal mixed effects model with climate action (WEPT), as the dependent variable, condition as the fixed effect, including country as random effects. Estimates are shown relative to the Control Condition.

Intervention	Estimate	SE	*z*	*d*	*p*
BindingMoral	0.04	0.04	0.89	0.03	0.375
SciConsens	0.03	0.04	0.65	-0.01	0.513
DynamicNorm	0.01	0.04	0.25	-0.01	0.800
SystemJust	-0.05	0.04	-1.39	-0.05	0.163
PluralIgnorance	-0.07	0.04	-1.82	-0.05	0.068
CollectAction	-0.11	0.04	-3.02	-0.06	**0.003**
FutureSelfCont	-0.12	0.04	-3.04	-0.06	**0.002**
LetterFutureGen	-0.23	0.04	-5.67	-0.13	**< 0.001**
WorkTogetherNorm	-0.25	0.04	-6.61	-0.15	**< 0.001**
PsychDistance	-0.27	0.04	-7.11	-0.15	**< 0.001**
NegativeEmotions	-0.29	0.04	-7.67	-0.16	**< 0.001**

Table S13: Coefficient table for an ordinal mixed effects model with climate action (i.e., number of trees planted in the WEPT) as the dependent variable, belief in climate change as the fixed effect, including country random effects.

Predictor	Estimate	SE	*z*	*p*
Belief	0.01	0.0003	29.84	**< .001**

Table S14: Coefficient table for an ordinal mixed effects model with climate action (i.e., number of trees planted in the WEPT) as the dependent variable, policy support as the fixed effect, including country random effects.

Predictor	Estimate	SE	*z*	*p*
Policy	0.01	0.0004	30.72	**< .001**

Table S15: Coefficient table for an ordinal mixed effects model with climate action (i.e., number of trees planted in the WEPT) as the dependent variable, willingness to share climate information as the fixed effect, including country random effects.

Predictor	Estimate	SE	*z*	*p*
Willingness to share	0.33	0.02	16.29	**< .001**

Table S16: Coefficient table for an ordinal mixed effects model with climate action (i.e., number of trees planted in the WEPT) as the dependent variable, belief in climate change in each condition as the fixed effect, including country random effects.

Belief:Intervention	Estimate	SE	*z*	*p*
Belief:SciConsens	0.012	0.0005	24.96	< **0.001**
Belief:BindingMoral	0.012	0.0005	24.77	< **0.001**
Belief:DynamicNorm	0.011	0.0005	24.30	< **0.001**
Belief:Control	0.011	0.0005	23.93	< **0.001**
Belief:SystemJust	0.011	0.0005	22.72	< **0.001**
Belief:PluralIgnorance	0.011	0.0005	22.40	< **0.001**
Belief:CollectAction	0.010	0.0005	21.46	< **0.001**
Belief:FutureSelfCont	0.010	0.0005	20.71	< **0.001**
Belief:WorkTogetherNorm	0.009	0.0005	19.21	< **0.001**
Belief:NegativeEmotions	0.008	0.0005	17.97	< **0.001**
Belief:LetterFutureGen	0.009	0.0005	17.85	< **0.001**
Belief:PsychDistance	0.008	0.0005	17.40	< **0.001**

Table S17: Coefficient table for an ordinal mixed effects model with climate action (i.e., number of trees planted in the WEPT) as the dependent variable, policy support in each condition as the fixed effect, including country random effects.

Policy:Intervention	Estimate	SE	*z*	*p*
Policy:SciConsens	0.015	0.0006	26.760	**< 0.001**
Policy:BindingMoral	0.015	0.0006	26.580	**< 0.001**
Policy:DynamicNorm	0.015	0.0006	25.980	**< 0.001**
Policy:Control	0.015	0.0006	25.850	**< 0.001**
Policy:SystemJust	0.014	0.0006	24.650	**< 0.001**
Policy:PluralIgnorance	0.014	0.0006	24.270	**< 0.001**
Policy:CollectAction	0.013	0.0005	23.550	**< 0.001**
Policy:FutureSelfCont	0.013	0.0006	22.370	**< 0.001**
Policy:WorkTogetherNorm	0.012	0.0006	20.590	**< 0.001**
Policy:LetterFutureGen	0.011	0.0006	20.000	**< 0.001**
Policy:NegativeEmotions	0.011	0.0006	19.880	**< 0.001**
Policy:PsychDistance	0.011	0.0006	19.200	**< 0.001**

Table S18: Coefficient table for an ordinal mixed effects model with climate action (i.e., number of trees planted in the WEPT) as the dependent variable, willingness to share information in each condition as the fixed effect, including country random effects.

Sharing:Intervention	Estimate	SE	*z-value*	*p*
Share:DynamicNorm	0.471	0.0454	10.390	**< 0.001**
Share:BindingMoral	0.466	0.0460	10.133	**< 0.001**
Share:SciConsens	0.422	0.0454	9.296	**< 0.001**
Share:Control	0.439	0.0475	9.242	**< 0.001**
Share:CollectAction	0.340	0.0442	7.678	**< 0.001**
Share:PluralIgnorance	0.357	0.0466	7.664	**< 0.001**
Share:SystemJust	0.326	0.0452	7.211	**< 0.001**
Share:FutureSelfCont	0.341	0.0493	6.913	**< 0.001**
Share:LetterFutureGen	0.243	0.0482	5.037	**< 0.001**
Share:WorkTogetherNorm	0.218	0.0438	4.976	**< 0.001**
Share:PsychDistance	0.158	0.0459	3.438	**< 0.001**
Share:NegativeEmotions	0.139	0.0430	3.239	**0.001**

Table S19: Coefficient table from analysis of belief. Results are from a linear mixed effects model with climate beliefs as the dependent variable, condition as the fixed effect, along with total condition time as a covariate, including item (4 beliefs), participant, and country as random effects. Estimates are shown relative to the Control Condition.

Intervention	Estimate	SE	*df*	*t*	*p*
(Intercept)	79.63	0.88	80.05	90.83	**< .001**
Condition Time	0.001	0.0002	59162.38	5.75	**< .001**
PsychDistance	3.08	0.47	59165.59	6.55	**< .001**
CollectAction	2.30	0.46	59165.43	5.01	**< .001**
SystemJust	1.90	0.46	59169.27	4.13	**< .001**
SciConsens	1.56	0.46	59168.19	3.40	**< .001**
FutureSelfCont	1.64	0.48	59175.38	3.39	**< .001**
LetterFutureGen	1.60	0.49	59169.22	3.26	**0.001**
BindingMoral	1.43	0.46	59171.14	3.08	**0.002**
PluralIgnorance	1.18	0.46	59160.52	2.56	**0.010**
DynamicNorm	1.00	0.46	59183.66	2.17	**0.030**
NegativeEmotions	0.73	0.46	59166.27	1.58	0.114
WorkTogetherNorm	-0.55	0.46	59159.98	-1.21	0.227

Table S20: Coefficient table from analysis of policy support. Results are from a linear mixed effects model with climate policy support, as the dependent variable, condition as the fixed effect, along with total condition time as a covariate, including item (9 policies), participant, and country as random effects. Estimates are shown relative to the Control Condition.

Intervention	Estimate	SE	*df*	*t*	*p*
(Intercept)	70.00	4.02	8.54	17.40	**< .001**
Condition Time	0.0008	0.0002	58259.82	4.64	**< .001**
CollectAction	2.92	0.37	58565.25	7.80	**< .001**
LetterFutureGen	2.62	0.40	58598.20	6.52	**< .001**
FutureSelfCont	1.96	0.40	58596.07	4.94	**< .001**
DynamicNorm	1.30	0.38	58596.74	3.45	**< .001**
PsychDistance	1.24	0.38	58642.02	3.24	**0.001**
SystemJust	1.04	0.38	58566.00	2.76	**0.006**
SciConsens	1.00	0.38	58602.11	2.67	**0.008**
BindingMoral	0.94	0.38	58574.93	2.48	**0.013**
PluralIgnorance	0.50	0.38	58573.00	1.32	0.188
WorkTogetherNorm	-0.05	0.37	58620.33	-0.14	0.890
NegativeEmotions	-0.27	0.37	58575.42	-0.72	0.474

Table S21: Coefficient table from analysis of Social Media Sharing. Results are from logistic mixed effects model with social media sharing as the dependent variable, condition as the fixed effect, along with total condition time as a covariate, including country as random effects. Estimates are shown relative to Control.

Intervention	Estimate	SE	*z*	*p*
(Intercept)	0.07	0.15	0.50	0.620
Condition Time	0.000007	0.00003	0.24	0.812
NegativeEmotions	0.48	0.05	9.43	**< .001**
CollectAction	0.40	0.05	7.76	**< .001**
LetterFutureGen	0.39	0.05	7.17	**< .001**
PsychDistance	0.35	0.05	6.61	**< .001**
DynamicNorm	0.31	0.05	6.11	**< .001**
FutureSelfCont	0.31	0.05	5.79	**< .001**
WorkTogetherNorm	0.23	0.05	4.66	**< .001**
SystemJust	0.23	0.05	4.61	**< .001**
BindingMoral	0.19	0.05	3.80	**< .001**
SciConsens	0.18	0.05	3.65	**< .001**
PluralIgnorance	0.10	0.05	1.92	0.055

Table S22: Coefficient table from analysis of WEPT. Results are from an ordinal mixed effects model with climate action (WEPT), as the dependent variable, condition as the fixed effect, along with intervention time as a covariate, including country as random effects. Estimates are shown relative to the Control Condition.

Intervention	Estimate	SE	*z*	*p*
Condition Time	0.001	0.000	18.88	**< .001**
BindingMoral	0.27	0.04	6.20	**< .001**
SciConsens	0.25	0.04	5.67	**< .001**
DynamicNorm	0.19	0.04	4.35	**< .001**
PluralIgnorance	0.12	0.04	2.86	**0.004**
SystemJust	0.09	0.04	2.13	**0.033**
CollectAction	-0.06	0.04	-1.52	0.129
WorkTogetherNorm	-0.18	0.04	-4.30	**< .001**
FutureSelfCont	-0.19	0.04	-4.31	**< .001**
NegativeEmotions	-0.29	0.04	-7.01	**< .001**
PsychDistance	-0.34	0.04	-8.30	**< .001**
LetterFutureGen	-0.38	0.04	-8.79	**< .001**

Table S23: Coefficient table from analysis of WEPT. Results are from an ordinal mixed effects model with climate action (WEPT), as the dependent variable, condition by condition time interaction as fixed effects, including country as random effects. Estimates are shown relative to the Control Condition.

Fixed Effect	Estimate	SE	*z*	*p*
Condition Time	0.002	0.000	8.572	< **.001**
SciConsens	0.448	0.076	5.892	< **.001**
DynamicNorm	0.418	0.077	5.447	< **.001**
PluralIgnorance	0.396	0.075	5.248	< **.001**
SystemJust	0.364	0.077	4.732	< **.001**
CollectAction	0.192	0.079	2.442	**0.015**
WorkTogetherNorm	0.179	0.079	2.269	**0.023**
FutureSelfCont	0.151	0.081	1.870	**0.061**
NegativeEmotions	0.050	0.079	0.626	.531
LetterFutureGen	-0.029	0.085	-0.341	.733
PsychDistance	-0.104	0.083	-1.262	.207
BindingMoral	-0.646	0.076	-8.501	< **.001**
BindingMoral:Condition Time	0.753	0.021	35.598	< **.001**
SciConsens:Condition Time	0.003	0.001	2.260	**.024**
DynamicNorm:Condition Time	-0.001	0.000	-1.269	.204
PluralIgnorance:Condition Time	-0.001	0.000	-2.562	**.010**
SystemJust:Condition Time	-0.001	0.000	-3.572	< **.001**
CollectAction:Condition Time	-0.001	0.000	-3.800	< **.001**
PsychDistance:Condition Time	-0.001	0.000	-4.072	< **.001**
LetterFutureGen:Condition Time	-0.001	0.000	-5.235	< **.001**

NegativeEmotions:Condition Time	-0.001	0.000	-5.246	**< .001**
FutureSelfCont:Condition Time	-0.001	0.000	-5.254	**< .001**
WorkTogetherNorm:Condition Time	-0.002	0.000	-5.594	**< .001**

Table S24: Coefficient table from analysis of WEPT. Results are from an ordinal mixed effects model with climate action (WEPT) as the dependent variable (coded as completed numbers of pages scored at least as 80% accurate), condition as the fixed effect, including country as random effects. Estimates are shown relative to the Control Condition.

Intervention	Estimate	SE	*z*	*p*
BindingMoral	0.03	0.04	0.89	0.375
SciConsens	0.02	0.04	0.65	0.513
DynamicNorm	0.01	0.04	0.25	0.800
SystemJust	-0.05	0.04	-1.39	0.163
PluralIgnorance	-0.07	0.04	-1.82	0.068
CollectAction	-0.11	0.04	-3.02	**0.003**
FutureSelfCont	-0.12	0.04	-3.04	**0.002**
LetterFutureGen	-0.23	0.04	-5.67	**< .001**
WorkTogetherNorm	-0.25	0.04	-6.61	**< .001**
PsychDistance	-0.27	0.04	-7.11	**< .001**
NegativeEmotions	-0.29	0.04	-7.67	**< .001**

Table S25: To test whether there was a main effect of condition (for each outcome variable), we conducted a Wald test for each outcome. The Wald test evaluated the null hypothesis that the joint distribution of the estimated coefficients for all intervention conditions was the same as the estimated coefficient for the control condition. The p-values below 0.001 for all tested outcomes indicate a significant divergence of the joint distribution of estimated coefficients from that of the control, suggesting that the interventions had varying effects.

Outcome	W - statistics	df	*p*
Belief	160.99	11	**< 0.001**
Policy Support	105.10	11	**< 0.001**
Social Media Sharing	154.26	11	**< 0.001**
WEPT	227.89	11	**< 0.001**
